# Income-Based Inequalities in Health System Performance in the US and South Korea

**DOI:** 10.1001/jamahealthforum.2026.0136

**Published:** 2026-03-20

**Authors:** Sungchul Park, Karen Eggleston, Young Kyung Do, David M. Cutler

**Affiliations:** 1 Department of Health Policy and Management, College of Health Science, Korea University, Seoul, South Korea; 2 Walter H. Shorenstein Asia-Pacific Research Center, Stanford University, Stanford, California; 3 National Bureau of Economic Research, Cambridge, Massachusetts; 4 Department of Health Policy and Management, College of Medicine, Seoul National University, Seoul, South Korea; 5 Institute of Health Policy and Management, Seoul National University Medical Research Center, Seoul, South Korea; 6 Department of Economics, Harvard University, Cambridge, Massachusetts; 7 Harvard Kennedy School, Cambridge, Massachusetts; 8 Harvard T.H. Chan School of Public Health, Harvard University, Boston, Massachusetts

## Abstract

**Importance:**

Income is a key social determinant of health, yet its influence on health system performance may differ across settings. Cross-national comparisons can help identify where income-related disparities are most pronounced and inform targeted policy responses; the US and South Korea are 2 members of the Organisation for Economic Co-operation and Development with high poverty rates but different health systems.

**Objective:**

To compare health system performance and income-related inequalities in health system performance between the US and South Korea.

**Design, Setting, and Participants:**

This repeated cross-sectional study including nationally representative samples of noninstitutionalized adults from the US and South Korea used data from the Medical Expenditure Panel Survey (MEPS; 2010-2019), National Health and Nutrition Examination Survey (NHANES; 2009-2018), Korean Health Panel Study (KHPS; 2010-2019), and Korean National Health and Nutrition Examination Survey (KNHANES; 2010-2019). Data were analyzed from March 2024 to March 2025.

**Exposures:**

Annual household income, categorized into country-specific deciles.

**Main Outcomes and Measures:**

The main outcomes were 30 indicators across 6 domains: health care spending, health care utilization, access to care, health status, behavioral risk factors, and clinical outcomes. To evaluate income-related inequalities in outcomes, adjusted mean values across income deciles were estimated using regression models.

**Results:**

The sample included 224 168 US adults (female: 51.1% in MEPS, 51.7% in NHANES) and 179 452 South Korean adults (female: 52.4% in KHPS, 56.1% in KNHANES). Mean (SD) age was 46.6 (18.0) years in MEPS, 46.5 (17.4) years in NHANES, 47.7 (16.2) years in KHPS, and 50.5 (17.1) years in KNHANES. US adults had higher mean total health care spending (lowest income decile: $7852 [95% CI, $7456-$8247]; highest decile: $6510 [95% CI, $6218-$6802]) than South Korean adults (lowest decile: $1184 [95% CI, $1105-$1263]; highest decile: $1025 [95% CI, $950-$1100]) despite similar levels of self-reported good health. A 1-decile increase in income was associated with a difference of −$142 (95% CI, −$179 to −$104) in total health care spending in the US compared with −$33 (95% CI, −$41 to −$25) in South Korea. A 1-decile increase in income was associated with an increase of 2.4 (95% CI, 2.3-2.5) percentage points (pp) in self-reported good health in the US compared with 1.5 (95% CI, 1.4-1.6) pp in South Korea. Income-related disparities in preventive service use were also larger in the US, ranging from 0.2 (95% CI, 0.2-0.2) pp for cervical cancer screening to 4.0 (95% CI, 3.9-4.1) pp for dental checkups. In South Korea, disparities ranged from 0.6 (95% CI, 0.4-0.8) pp for dental checkups to 2.0 (1.8-2.2) pp for routine checkups. Similar income gradients were observed in access to care and behavioral risk factors. Differences in clinical outcomes were modest in both countries.

**Conclusions and Relevance:**

In this cross-sectional study, income was associated with disparities in health system performance in both the US and South Korea, with larger differences by income in the US. The findings suggest that structural and systemic policy efforts are needed to address income-based health inequalities, particularly in the US.

## Key Points

**Question:**

How do income-related inequalities in health system performance differ between the US and South Korea?

**Findings:**

In this cross-sectional study of 224 168 US adults and 179 452 South Korean adults, higher-income adults had lower health care spending, greater preventive care use, greater access to care, fewer behavioral risk factors, and better self-reported health than lower-income adults in both countries. These inequalities were generally more pronounced in the US.

**Meaning:**

The findings suggest that income is associated with disparities in health system performance in the US and South Korea, with larger differences by income in the US.

## Teaser

This cross-sectional study compares health system performance by household income in the US and South Korea.

## Introduction

Income inequality poses a significant challenge to health systems, contributing to persistent disparities in health.^1,2,3,4,5,6,7,8^ The relationship between income and health is shaped by complex and multidimensional mechanisms, including unequal access to care, disparities in the quality of services received, and greater exposure to adverse social determinants. These inequalities are often embedded within structural features of health systems.^2^

The US and South Korea offer distinct yet instructive cases for examining how income inequality interacts with health system performance. Both countries have substantial income-related inequalities. In 2022, the US recorded the second-highest poverty rate (18%) among member countries in the Organisation for Economic Co-operation and Development, while South Korea reported the highest poverty rate among older individuals (age, ≥65 years) (38%).^9^ Life expectancy at birth is higher in South Korea than in the US (84.5 years vs 76.4 years),^9^ but income-related gaps remain in both settings.^10,11,12,13,14,15^ The life expectancy gap between the highest and lowest income quintiles is 7.91 years for men and 4.34 years for women in South Korea^13^ and 9.25 years for men and 5.87 years for women in the US.^10^

While both countries face similar equity challenges, the magnitude and patterns of income-related inequalities may differ, partly due to differences in health system structures. In the US, care is delivered through a fragmented, market-based system with limited public coverage, wide variation in benefits, and high out-of-pocket costs. These structural features place a disproportionate financial burden on low-income populations and can magnify disparities in access, utilization, and health outcomes. By contrast, South Korea offers universal health care coverage through the single-payer National Health Insurance program, ensuring broad access to a standardized set of services. However, high out-of-pocket costs, particularly for services not fully covered by National Health Insurance, remain a barrier to equitable access. Out-of-pocket spending accounted for 29% of total health care expenditure in South Korea in 2022, exceeding both the US (11%) and the Organisation for Economic Co-operation and Development mean (18%).^16^

Although income-related inequalities in health care and health have been widely documented within individual countries,^17,18,19,20,21,22,23,24^ 2 important gaps remain. First, most existing studies have focused on only a small number of specific outcomes, limiting a comprehensive understanding of inequalities across the full spectrum of health system performance. Second, few studies have conducted systematic cross-national comparisons, which are essential to understanding how policy and institutional contexts shape the magnitude and nature of inequalities. Addressing these gaps is critical to identifying where income-related inequalities are most pronounced and informing targeted policy responses.

To address these gaps, we conducted a systematic comparison of health system performance and income-based inequalities in health system performance between the US and South Korea. Using nationally representative datasets, we examined 30 indicators across 6 key domains: health care spending, health care utilization, access to care, health status, behavioral risk factors, and clinical outcomes. Each domain captures a distinct yet interconnected dimension of health system performance. Investigating these domains collectively provides a multidimensional view of inequalities, highlighting areas where inequalities are most pronounced.

## Methods

This cross-sectional study was reviewed and approved by the institutional review board of Korea University. Patient consent was waived because deidentified data were used. The study adhered to the Strengthening the Reporting of Observational Studies in Epidemiology (STROBE) reporting guideline.

### Data

We conducted a repeated cross-sectional analysis using nationally representative survey data of noninstitutionalized adults from the US and South Korea. US data were drawn from the Medical Expenditure Panel Survey (MEPS; 2010-2019) and the National Health and Nutrition Examination Survey (NHANES; 2009-2018). Korean data were obtained from the Korean Health Panel Study (KHPS; 2010-2019) and the Korean National Health and Nutrition Examination Survey (KNHANES; 2010-2019).

### Sample

We first identified noninstitutionalized adults aged 18 years or older from each dataset. The sample was then categorized into income deciles based on annual household income, defined consistently across datasets as the total income from all sources received by all household members. To enable meaningful within-country comparisons and account for cross-country differences in income distribution, we followed prior studies by using relative income measures.^10,12^ Since the NHANES dataset reports only income deciles rather than actual income amounts, comparing absolute income levels was not possible. Therefore, income deciles were constructed separately for each country and used as the primary independent variable.

### Outcomes

We assessed 30 indicators across 6 domains. First, health care spending was defined as total annual expenditures on inpatient, outpatient, and emergency department services and prescription medications. Both overall and service-specific spending were analyzed. All spending estimates were inflation adjusted using the gross domestic product deflator and are expressed in 2021 US dollars. Second, health care utilization was evaluated using 2 approaches: service volume, defined as the number of inpatient admissions, outpatient visits, and emergency department visits per 1000 individuals, and receipt of preventive services. Third, access to care was evaluated using 3 measures: having a usual source of care, experiencing any unmet need for medical care, and reporting unmet needs specifically due to cost. Fourth, health status was measured using self-reported good health. Fifth, behavioral risk factors included smoking history, current smoking status, overweight and obesity, and excessive alcohol consumption. Sixth, clinical outcomes were assessed using survey and biomarker-based indicators. Detailed definitions and data sources for all measures are provided in eTable 1 in Supplement 1.

### Statistical Analysis

We compared the distributions of annual household income and demographic characteristics between the US and South Korea. To evaluate income-related inequalities in outcomes, we estimated adjusted mean values across income deciles using regression models that controlled for age; sex; age squared; the interaction between age, age squared, and sex; and year fixed effects. Consistent with the Institute of Medicine framework,^25^ which defines health care disparities as differences in care quality not explained by health care needs or preferences, we limited covariate adjustment to demographic factors to avoid underestimating structural inequities. Using the regression models, we calculated adjusted mean values for both the lowest and the highest income deciles, holding all other variables constant. We also quantified income-related disparities as the change in each outcome associated with a 1-decile increase in household income. Analyses were conducted separately for each country. Continuous outcomes were analyzed using 2-part models for health care spending and utilization (logistic regression and a generalized linear model with log link and gamma family). Binary outcomes were analyzed using linear probability models. To assess the extent of income-related disparities between the US and South Korea, we conducted a regression analysis including income decile and an interaction term between country and income decile while controlling for the variables used in the previous analysis. The interaction term was used to evaluate whether income-related disparities differed significantly between the 2 countries.

We also conducted several sensitivity analyses. First, to account for potential changes following the implementation of the Affordable Care Act, we repeated the analysis using data from 2014 to 2019. Second, because income variation differs by age, we conducted separate analyses for individuals aged 18 to 64 years, 65 years or older, and 40 years or older. Survey weights were applied to ensure that the sample characteristics were representative of each population. Analyses were performed using Stata, version 17.0 (StataCorp LLC). Data were analyzed from March 2024 to March 2025.

## Results

### Descriptive Statistics

The sample included 224 168 US adults (197 002 from MEPS and 27 166 from NHANES) and 179 452 South Korean adults (115 070 from KHPS and 64 382 from KNHANES) (eTable 2 in Supplement 1). In the MEPS sample, 51.1% of individuals were female and 48.8% were male; mean (SD) age was 46.6 (18.0) years. In the NHANES sample, 51.7% of individuals were female and 48.2% were male; mean (SD) age was 46.5 (17.4) years. In the KHPS sample, 52.4% of individuals were female and 47.6% were male; mean (SD) age was 47.7 (16.2) years. In the KNHANES sample, 56.1% of individuals were female and 43.8% were male; mean (SD) age was 50.5 (17.1) years. The age distributions differed between countries; the US had a larger proportion of adults younger than 39 years, whereas South Korea had a higher proportion of adults aged 50 to 69 years (eFigure 1 in Supplement 1). Unadjusted outcome measures are reported in eTable 3 in Supplement 1.

**Figure 1.**
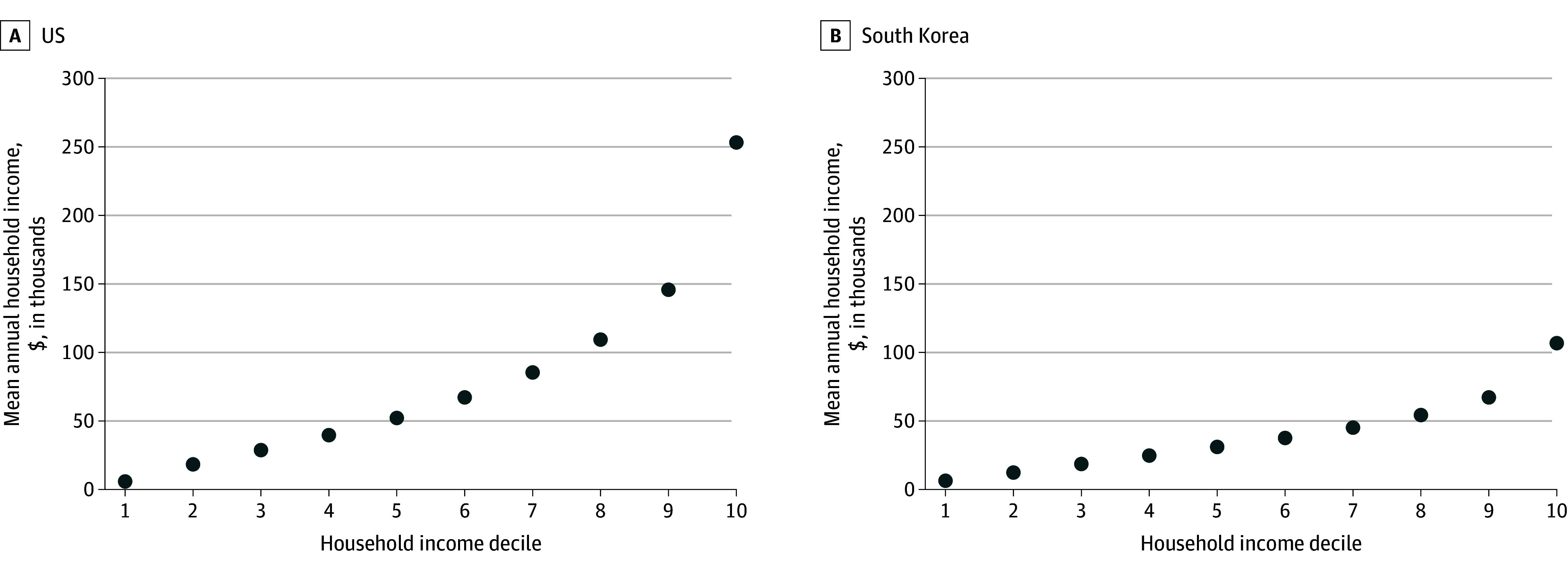
Dot Plot of Household Income Distribution Among Adults in the US and South Korea Annual household income data were obtained from the 2010 to 2019 Medical Expenditure Panel Survey for the US and the 2010 to 2019 Health Panel Survey for South Korea. Income was adjusted for inflation using gross domestic product deflators and converted to US dollars based on 2021 values.

Substantial income inequality was observed in both countries, but the inequalities were more pronounced in the US (Figure 1). In 2019, annual household income in the highest decile in the US was approximately 42 times greater than in the lowest decile in the US (mean [SD], $251 531 [$79 628] vs $5923 [$4758]) compared with a 16-fold difference in South Korea (mean [SD], $117 654 [$61 249] vs $7375 [$2078]).

### Health Care Spending

**Figure 2.**
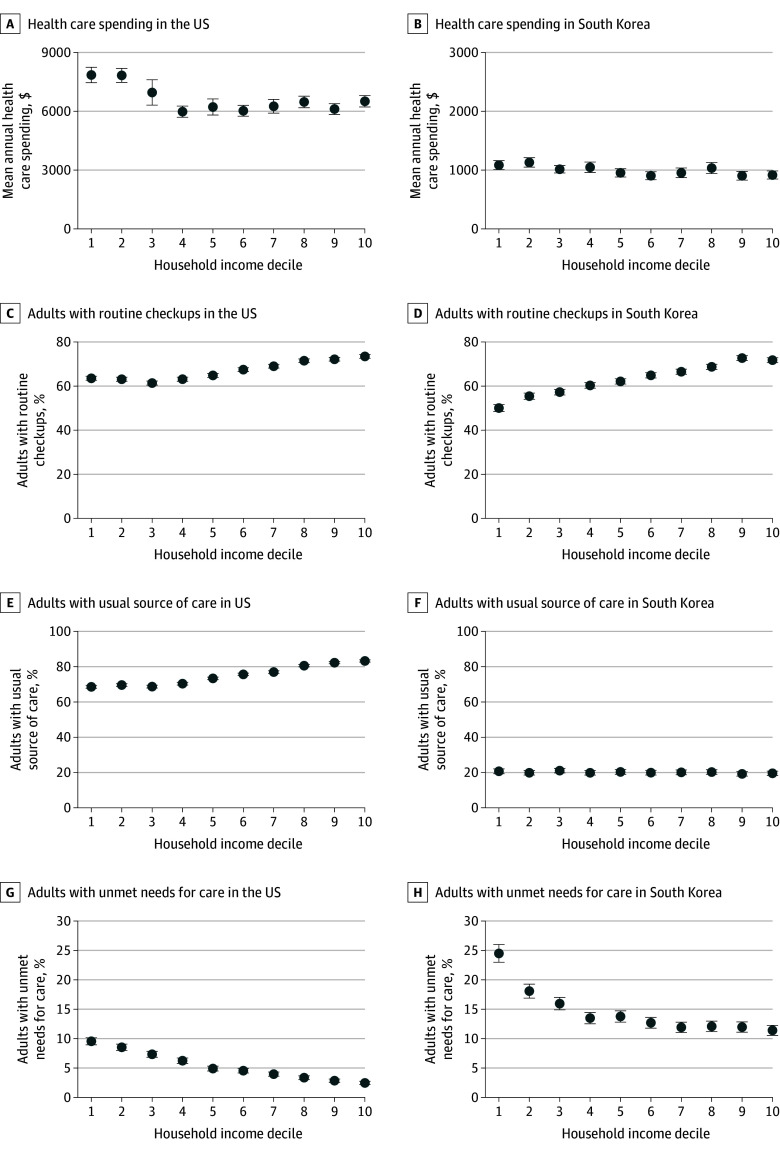
Dot Plots of Health Care Spending, Utilization, and Access by Household Income Decile Among Adults in the US and South Korea Regression models are described in the Statistical Analysis subsection of the Methods section. Survey weights were applied to make the sample characteristics representative of each population. Values for South Korea were reestimated assuming an age-sex distribution equivalent to that of the US. Error bars represent 95% CIs.

Overall, adults in the US had higher total health care spending than adults in South Korea, but in both countries, higher-income adults had lower total health care spending than lower-income adults (Table 1, Figure 2A and B, and eFigure 2 in Supplement 1). Among adults in the lowest and highest income deciles, adjusted mean total spending in the US was $7852 (95% CI, $7456-$8247) and $6510 (95% CI, $6218-$6802), respectively, compared with $1184 (95% CI, $1105-$1263) and $1025 (95% CI, $950-$1100), respectively, in South Korea. These disparities were more pronounced in the US in absolute terms but similar to South Korea as a percentage of mean health care spending; each 1-decile increase in annual household income was associated with a difference of −$142 (95% CI, −$179 to −$104), or −2%, in total health care spending compared with −$33 (95% CI, −$41 to −$25), or −2%, in South Korea. The between-country difference was statistically significant (eTable 4 in Supplement 1).

**Table 1.** Income Inequalities in Health Care Spending, Utilization, and Access to Care Among Adults in the US and South Korea

Outcome	US			South Korea		
	Adjusted value (95% CI)^a^^,^^b^		Change per 1-decile increase in income (95% CI)^a^^,^^c^	Adjusted value (95% CI)^a^^,^^b^		Change per 1-decile increase in income (95% CI)^a^^,^^c^
	Lowest income decile	Highest income decile		Lowest income decile	Highest income decile	
**Health care spending, mean, $^d^**						
Total	7852 (7456 to 8247)	6510 (6218 to 6802)	−142 (−179 to −104)	1184 (1105 to 1263)	1025 (950 to 1100)	−33 (−41 to −25)
Inpatient admissions	2497 (2275 to 2718)	1501 (1311 to 1691)	−98 (−121 to −75)	518 (461 to 576)	355 (296 to 414)	−24 (−31 to −18)
Outpatient visits	1543 (1458 to 1627)	1946 (1864 to 2029)	49 (39 to 59)	627 (597 to 656)	639 (603 to 674)	−8 (−12 to −5)
Emergency department visits	368 (337 to 399)	216 (193 to 238)	−13 (−16 to −9)	12 (10 to 15)	10 (8 to 12)	0 (−1 to 0)
**Health care utilization**						
Type of visit, mean, per 1000 people						
Inpatient admissions	164 (155 to 174)	71 (65 to 77)	−9.2 (−10.0 to −8.3)	236 (218 to 253)	156 (139 to 172)	−8.6 (−10.2 to −7.1)
Outpatient visits	7516 (7204 to 7829)	7290 (7088 to 7493)	30.5 (2.5 to 58.5)	25 109 (24 315 to 25903)	18 021 (17 447 to 18 594)	−665.6 (−722.4 to −608.7)
Emergency department visits	384 (367 to 401)	111 (104 to 118)	−26.4 (−27.7 to −25.1)	116 (103 to 129)	98 (88 to 109)	−3.3 (−4.4 to −2.2)
Preventive care, %						
Routine checkup	63.5 (62.6 to 64.4)	73.4 (72.6 to 74.3)	1.4 (1.3 to 1.5)	50 (48.5 to 51.6)	71.8 (70.7 to 72.9)	2 (1.8 to 2.2)
Dental checkup	24 (23.2 to 24.9)	57.1 (56.2 to 57.9)	4 (3.9 to 4.1)	72.4 (70.4 to 74.4)	79.4 (78 to 80.7)	0.6 (0.4 to 0.8)
Influenza vaccination	52.5 (51 to 54.1)	64.7 (63.4 to 65.9)	1.4 (1.2 to 1.5)	50 (48.2 to 51.8)	51.1 (49.2 to 52.9)	−0.1 (−0.3 to 0.1)
Breast cancer screening	66.7 (64.6 to 68.9)	82.4 (80.8 to 84)	2.1 (1.9 to 2.3)	50.1 (46 to 54.3)	69.2 (64.5 to 74.0)	1.4 (0.8 to 2.1)
Colorectal cancer screening	47.5 (45.8 to 49.2)	69.7 (68.4 to 70.9)	2.6 (2.4 to 2.7)	47.9 (44.5 to 51.3)	71.4 (68.0 to 74.7)	1.9 (1.5 to 2.4)
Cervical cancer screening	88.6 (88.1 to 89.2)	90.3 (89.5 to 91)	0.2 (0.2 to 0.2)	95.5 (94.7 to 96.2)	98 (97.8 to 98.2)	−0.1 (−0.1 to 0)
**Access to care, %**						
Having a usual source of care	68.6 (67.7 to 69.4)	83.2 (82.6 to 83.9)	1.8 (1.7 to 1.9)	20.5 (19.2 to 21.9)	19.2 (18.0 to 20.5)	−0.2 (−0.4 to −0.1)
Unmet need for medical care	9.5 (8.9 to 10.1)	2.5 (2.2 to 2.7)	−0.7 (−0.8 to −0.7)	24.1 (22.7 to 25.6)	11.3(10.5 to 12.2)	−0.8 (−0.9 to −0.7)
Unmet need for medical care due to costs	6.6 (6.1 to 7.1)	0.4 (0.3 to 0.5)	−0.7 (−0.7 to −0.6)	11.6 (10.4 to 12.8)	0.5 (0.3 to 0.7)	−0.9 (−0.9 to −0.8)

^a^ Regression models are described in the Statistical Analysis subsection of the Methods section.

^b^ Survey weights were applied to ensure that the sample characteristics were representative of each population.

^c^ For preventive care and access to care, percentage points are shown.

^d^ Health care spending was adjusted for inflation using gross domestic product deflators and converted to US dollars based on 2021 values.

Spending patterns differed across types of care and countries. Inpatient and emergency department spending declined with increasing income in both countries, with steeper gradients in the US. Each 1-decile increase in income was associated with differences of −$98 (95% CI, −$121 to −$75) in inpatient spending and −$13 (95% CI, −$16 to −$9) in emergency department spending in the US compared with differences of −$24 (95% CI, −$31 to −$18) and $0 (95% CI, −$1 to $0) in South Korea. In contrast, outpatient spending followed divergent trends. In the US, each 1-decile increase in income was associated with an increase of $49 (95% CI, $39-$59) in outpatient spending, whereas in South Korea, each 1-decile increase in income was associated with a difference of −$8 (95% CI, −$12 to −$5).

### Health Care Utilization

Health care utilization was consistently lower in the US than in South Korea across all income levels, but in both countries, higher-income adults generally used fewer health care services than lower-income adults (Table 1 and eFigure 3 in Supplement 1). These income-related disparities were more pronounced in the US. Each 1-decile increase in household income was associated with differences of −9.2 (95% CI, −10.0 to −8.3) inpatient admissions per 1000 individuals and −26.4 (95% CI, −27.7 to −25.1) emergency department visits per 1000 individuals in the US compared with differences of −8.6 (95% CI, −10.2 to −7.1) inpatient admissions per 1000 individuals and −3.3 (95% CI, −4.4 to −2.2) emergency department visits per 1000 individuals in South Korea. For outpatient visits, income-related trends diverged. In the US, each 1-decile increase in income was associated with an increase of 30.5 (95% CI, 2.5-58.5) outpatient visits per 1000 individuals, whereas in South Korea, each 1-decile increase in income was associated with a difference of −665.6 (95% CI, −722.4 to −608.7) outpatient visits per 1000 individuals.

Preventive care use had mixed patterns across countries. At the country level, adults in the US reported higher utilization of routine checkups, influenza vaccinations, and breast cancer screenings, while South Korean adults had higher utilization of dental checkups, colorectal cancer screenings, and cervical cancer screenings. Notably, income-related disparities in preventive service use were wider in the US, ranging from 0.2 (95% CI, 0.2-0.2) percentage points (pp) for cervical cancer screening to 4.0 (95% CI, 3.9-4.1) pp for dental checkups. In contrast, these gaps were narrower in South Korea, ranging from 0.6 (95% CI, 0.4-0.8) pp for dental checkups to 2.0 (95% CI, 1.8-2.2) pp for routine checkups.

### Access to Care

In both countries, higher-income adults generally had greater access to care than lower-income adults; however, despite a higher number of visits per capita in South Korea, there was not better access to care (Table 1, Figure 2E-H, and eFigure 4 in Supplement 1). Specifically, the proportion of adults reporting a usual source of care was substantially higher in the US, at 68.6% (95% CI, 67.7%-69.4%) in the lowest income decile and 83.2% (95% CI, 82.6%-83.9%) in the highest income decile compared with 20.5% (95% CI, 19-2%-21.9%) and 19.2% (95% CI, 18.0%-20.5%), respectively, in South Korea. Similarly, reports of unmet medical needs were less common in the US (9.5% [95% CI, 8.9%-10.1%] and 2.5% [95% CI, 2.2% to 2.7%] in the lowest and highest income deciles, respectively) than in South Korea (24.1% [95% CI, 22.7% to 25.6%] and 11.3% [95% CI, 10.5 to 12.2], respectively), as were reports of unmet needs due to cost (US: 6.6% [95% CI, 6.1%-7.1%] and 0.4% [95% CI, 0.3%-0.5%], respectively; South Korea: 11.6% [95% CI, 10.4%-12.8%] and 0.5% [95% CI, 0.3%-0.7%, respectively).

When examining income-related disparities across the income distribution, each 1-decile increase in income was associated with a difference of 1.8 (95% CI, 1.7-1.9) pp in reporting a usual source of care in the US but −0.2 (95% CI, −0.4 to −0.1) pp in South Korea. In contrast, income gradients in unmet medical needs were comparable; each 1-decile increase in income was associated with a difference of −0.7 (95% CI, −0.8 to −0.7) pp in the US and −0.8 (95% CI, −0.9 to −0.7) pp in South Korea. Similarly, for unmet needs due to cost, each 1-decile increase in income was associated with a difference of −0.7 (95% CI, −0.7 to −0.6) pp in the US and −0.9 (95% CI, −0.9 to −0.8) pp in South Korea. South Korean adults in the lowest 2 deciles of income reported higher unmet needs due to costs than US adults in the lowest decile (eFigure 4 in Supplement 1).

### Health Status

**Figure 3.**
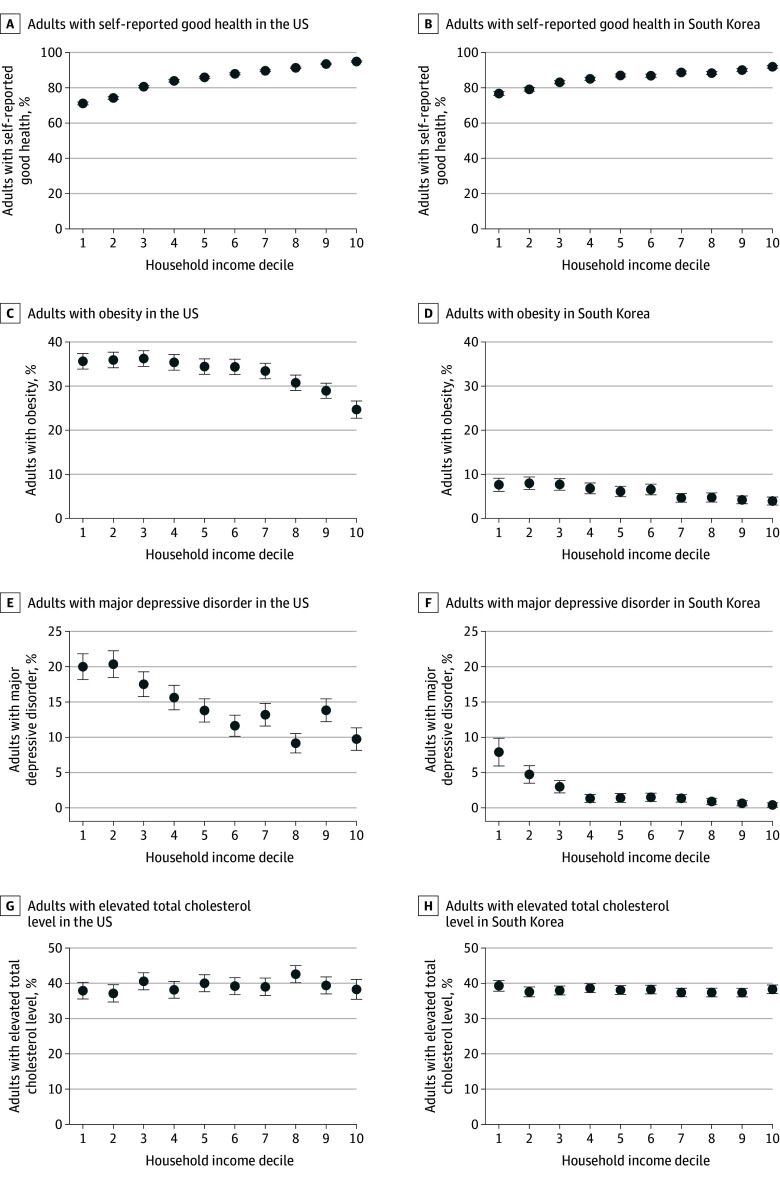
Dot Plots of Health Status, Risk Factors, and Clinical Outcomes by Household Income Decile Among Adults in the US and South Korea Regression models are described in the Statistical Analysis subsection of the Methods section. Survey weights were applied to make the sample characteristics representative of each population. The values for South Korea were reestimated assuming an age-sex distribution equivalent to that of the US. Error bars represent 95% CIs.

Self-reported health status was similar between the US and South Korea, but higher-income adults reported better health than lower-income adults in both countries (Table 2 and Figure 3A and B). In the US, 71.1% (95% CI, 70.3%-71.9%) of adults in the lowest income decile reported good health compared with 94.9% (95% CI, 94.5%-95.3%) in the highest income decile. In South Korea, the corresponding proportions were 76.9% (95% CI, 75.8%-78.1%) and 92.5% (95% CI, 91.3%-92.8%). Income-related disparities in self-reported health were slightly more pronounced in the US. Each 1-decile increase in household income was associated with an increase of 2.4 (95% CI, 2.3-2.5) pp in the likelihood of reporting good health in the US compared with 1.5 (95% CI, 1.4-1.6) pp in South Korea.

**Table 2.** Income Inequalities in Health Status, Risk Factors, and Clinical Outcomes Among Adults in the US and South Korea

Outcome	US			South Korea		
	Adjusted value, % (95% CI)^a^^,^^b^		Change per 1-decile increase in income, pp (95% CI)^a^	Adjusted value, % (95% CI)^a^^,^^b^		Change per 1-decile increase in income, pp (95% CI)^a^
	Lowest income decile	Highest income decile		Lowest income decile	Highest income decile	
Self-reported good health	71.1 (70.3 to 71.9)	94.9 (94.5 to 95.3)	2.4 (2.3 to 2.5)	76.9 (75.8 to 78.1)	92.5 (91.3 to 92.8)	1.5 (1.4 to 1.6)
Risk factors						
Ever smoker	53.4 (51.6 to 55.2)	27.2 (25.2 to 29.2)	−2.9 (−3.2 to −2.7)	47.1 (45.9 to 48.2)	34.2 (33.3 to 35.1)	−1.1 (−1.2 to −0.9)
Current smoker	28.6 (27.3 to 30.0)	3.3 (1.8 to 4.8)	−3.0 (−3.2 to −2.7)	28.9 (27.7 to 30.1)	14.7 (13.8 to 15.5)	−1.3 (−1.4 to −1.2)
Overweight	28.7 (27.0 to 30.5)	36.2 (34.2 to 38.1)	0.6 (0.3 to 0.9)	27.5 (24.9 to 30.1)	26.0 (24.1 to 27.9)	−0.1 (−0.3 to 0.2)
Obesity	35.6 (33.9 to 37.4)	24.7 (22.7 to 26.6)	−1.2 (−1.5 to −1.0)	7.6 (6.1 to 9.1)	3.9 (3.0 to 4.8)	−0.4 (−0.6 to −0.3)
Excessive alcohol consumption	17.4 (15.6 to 19.3)	8.1 (5.4 to 10.7)	−0.4 (−0.7 to −0.1)	16.6 (15.2 to 17.9)	12.3 (11.4 to 13.2)	−0.4 (−0.6 to −0.3)
Clinical outcomes						
Major depressive disorder	20.0 (18.2 to 21.8)	9.7 (8.2 to 11.3)	−1.1 (−1.3 to −0.9)	7.9 (5.9 to 9.9)	0.4 (0.1 to 0.7)	−0.5 (−0.6 to −0.4)
Uncontrolled hypertension (systolic)	34.0 (32.0 to 36.1)	22.5 (20.1 to 24.9)	−1.1 (−1.3 to −0.8)	27.5 (26.1 to 28.9)	22.2 (21.3 to 23.2)	−0.6 (−0.7 to −0.4)
Uncontrolled hypertension (diastolic)	23.5 (21.6 to 25.5)	21.9 (19.3 to 24.4)	−0.3 (−0.6 to −0.1)	39.3 (37.8 to 40.7)	37.4 (36.2 to 38.6)	−0.1 (−0.3 to 0)
Uncontrolled diabetes	12.1 (10.7 to 13.4)	5.6 (4.3 to 7.0)	−0.8 (−0.9 to −0.6)	11.8 (10.6 to 13.0)	7.7 (7.1 to 8.4)	−0.4 (−0.5 to −0.3)
Elevated total cholesterol level	37.9 (35.5 to 40.2)	38.2 (35.5 to 41.0)	0.2 (−0.1 to 0.5)	39.3 (37.7 to 40.9)	38.3 (37.1 to 39.5)	−0.1 (−0.3 to 0)
Low HDL cholesterol level	24.6 (22.5 to 26.8)	16.4 (14.2 to 18.5)	−1.1 (−1.3 to −0.9)	21.5 (20.1 to 22.8)	16.0 (15.1 to 17)	−0.4 (−0.6 to −0.3)
Elevated LDL cholesterol level	27.6 (24.5 to 30.8)	25.9 (22.2 to 29.6)	−0.2 (−0.6 to 0.2)	33.4 (30.5 to 36.3)	30.0 (27.7 to 32.3)	−0.3 (−0.6 to 0)
Hypertriglyceridemia	25.9 (22.9 to 28.9)	19.9 (16.6 to 23.3)	−0.9 (−1.3 to −0.5)	33.5 (32.0 to 35.0)	25.0 (23.9 to 26.1)	−0.7 (−0.8 to −0.5)

Abbreviations: HDL, high-density lipoprotein; LDL, low-density lipoprotein; pp, percentage points.

^a^ Regression models are described in the Statistical Analysis subsection of the Methods section.

^b^ Survey weights were applied to ensure that the sample characteristics were representative of each population.

### Behavioral Risk Factors

Behavioral risk factor patterns differed between the 2 countries (Table 2, Figure 3C and D, and eFigure 5 in Supplement 1). Adults in the US had a higher prevalence of overweight and obesity, while adults in South Korea were more likely to report current smoking and excessive alcohol consumption. In both countries, these risk factors were more common among lower-income adults, with income-related disparities more pronounced in the US. Each 1-decile increase in income was associated with differences of −3.0 (95% CI, −3.2 to −2.7) pp in current smoking, −1.2 (95% CI, −1.5 to −1.0) pp in obesity, and −0.4 (95% CI, −0.7 to −0.1) pp in excessive drinking in the US compared with differences of −1.3 (95% CI, −1.4 to −1.2) pp in current smoking and −0.4 (95% CI, −0.6 to −0.3) pp in obesity in South Korea; there was no difference in excessive drinking in South Korea (−0.1 pp; 95% CI, −0.3 to 0.2 pp).

### Clinical Outcomes

Patterns of clinical outcomes varied between the US and South Korea (Table 2, Figure 3E-H, and eFigure 6 in Supplement 1). Adults in the US had a higher prevalence of major depressive disorder, while adults in South Korea had higher rates of uncontrolled diastolic hypertension, elevated low-density lipoprotein cholesterol level, and hypertriglyceridemia. Other clinical indicators showed similar patterns across the 2 countries. Income-related disparities in clinical outcomes were present although generally modest in magnitude in both countries. In the US, disparities ranged from −1.1 (95% CI, −1.3 to −0.9) pp for major depressive disorder to −0.3 (95% CI, −0.6 to −0.1) pp for uncontrolled diastolic hypertension. In South Korea, disparities ranged from −0.7 (95% CI, −0.8 to −0.5) pp for hypertriglyceridemia to −0.1 (95% CI, −0.3 to 0) pp for uncontrolled diastolic hypertension.

### Between-Country Differences

The differences between countries were statistically significant for all measures except clinical outcomes (eTable 4 in Supplement 1). Income-related disparities in the US, compared with South Korea, were especially pronounced for health care spending, while differences for other outcomes were relatively modest.

### Sensitivity Analysis

The main findings generally remained robust across sensitivity analyses (eTable 5-12 in Supplement 1). However, compared with younger adults, disparities were wider among those aged 65 years or older in South Korea and similar or narrower among US adults aged 65 years or older.

## Discussion

Our cross-national comparison of health system performance between the US and South Korea demonstrated that despite substantially higher per capita health care spending in the US, overall health status and clinical outcomes were broadly similar between the 2 countries. Although the measures included in our analysis indicated comparable health outcomes, broader population health indicators, such as life expectancy at birth, remain lower in the US.^9^ This finding aligns with prior research indicating that higher spending in the US does not necessarily translate into better health outcomes, pointing to inefficiencies in care delivery.^26^

The US had worse outcomes than South Korea in several domains. Achieving similar clinical outcomes despite substantially higher spending reflects inefficiency in the US health system. One key contributor to the spending gap may be higher prices, likely stemming from limited regulation and competition and greater administrative expenses. We found that despite higher spending in the US, overall utilization in the US was not greater than that in South Korea, underscoring the need for greater consideration of price regulation and measures to increase transparency.^27^ Another contributor may be unfavorable social and behavioral risk factors rooted in broader structural inequalities outside the health care system that may influence population health outcomes. However, the US had more favorable outcomes than South Korea on multiple measures of access and care coordination, with these advantages evident even among low-income populations. These results suggest that certain components of the US delivery system function effectively.

South Korea, by contrast, had comparable health outcomes at lower per-capita spending, consistent with prior research.^28^ Although this performance reflects strengths of the universal health insurance system, it cannot be fully attributed to health system factors, as South Korea did not have more favorable outcomes than the US across all measures. South Korea also faces challenges in health care delivery.^29^ Despite universal coverage, unmet medical needs remain high, largely due to high out-of-pocket costs and limited benefit coverage. The low rate of having a usual source of care should be interpreted with caution, as adults in South Korea had substantially higher outpatient visit rates. This pattern may reflect broad and flexible access to care rather than limited access to a regular practitioner. However, freedom of practitioner choice, including direct access to specialists without primary care coordination, may contribute to fragmented care and reduced emphasis on prevention. The primary care workforce is also comparatively limited, with 23.9 primary care physicians per 100 000 people in South Korea vs 67.2 in the US.^30,31^ Consistent with these structural factors, preventive care measures were less favorable in South Korea. Strengthening primary care through incentive-aligned first-contact care, referral pathways, and payment reforms could enhance coordination and prevention while preserving patient choice.

Furthermore, our analysis revealed consistent patterns of income-related health inequalities across multiple domains in both countries, with disparities significantly more pronounced in the US. The higher level of income inequality in the US than South Korea may have contributed to more persistent gaps in access and utilization. In the US, where access remains closely tied to the ability to pay, income inequality was associated with disparities in coverage, access, and service use. Lower-income adults are more likely to be uninsured,^32^ and even those with insurance often face substantial out-of-pocket costs that discourage needed care.^33,34^ Notably, income-related disparities in health care spending were largest in the lowest income decile, while spending remained relatively stable across higher deciles. Higher spending in the lowest decile may reflect greater health needs and lower cost-sharing associated with Medicaid enrollment. In South Korea, income-related inequalities were smaller but still present despite universal coverage. High cost-sharing and limited benefit packages continue to impose financial barriers, indicating that income can still shape health care access and use within a universal insurance system. Nonetheless, the smaller income gradient may highlight the more protective role of universal coverage in mitigating disparities.

Beyond the health care system, health shapes income over the life course, and health outcomes are shaped through broader social and environmental pathways.^2,5^ Prior research has identified barriers, such as health care practitioner shortages, geographic isolation from medical facilities, and limited access to transportation in low-income communities, as key obstacles to timely and effective care.^2,5^ These structural disadvantages potentially amplify the reach and severity of income-related health disparities. Nevertheless, consistent with prior studies,^17,35,36,37^ we found that income-related disparities in clinical outcomes were present but generally modest in both countries. This finding may be due to the longer time horizon over which clinical outcomes develop. In addition, the availability of standardized treatment guidelines, public medication programs, and compensatory behaviors or informal support networks may help mitigate income-related differences in disease management.

### Limitations

Our study has several limitations. First, the study sample was limited to the noninstitutionalized population. Although the proportion of institutionalized adults is similar in both countries, differences in long-term care systems and population age structures may affect comparability. For example, because the proportion of institutionalized adults in South Korea includes a broader range of facilities and applies to an older population, a slightly larger share of individuals with medical complexity may be excluded from South Korean surveys. Second, our measures relied on self-reported data, which may introduce reporting errors. While multiple validation procedures were applied, these efforts may not fully mitigate all inaccuracies. Third, although most indicators were harmonized across datasets using consistent operational definitions, differences in the design and measurement of some indicators may limit cross-national comparability. Fourth, we were unable to account for all potential confounding factors; therefore, the findings should be interpreted as associations and not the causal effects of income. Fifth, our measure of health care spending did not include certain components, such as long-term care, which may lead to underestimation of total spending. In relation to this, our health status indicators captured only a subset of potential measures, and our outcomes did not include quality-of-care or process indicators due to data limitations; therefore, the findings may not fully generalize to broader outcomes, such as life expectancy or mortality, and may provide an incomplete picture of cross-national differences. Sixth, we assumed that the outcomes included in this study were primarily attributable to health system performance, but some outcomes may be more strongly influenced by factors outside the health care system. Seventh, household income was used as the primary socioeconomic measure, but asset information was unavailable, potentially underestimating individuals’ overall economic resources.

## Conclusions

This cross-sectional study found that despite greater per-capita health care spending in the US, overall health status and clinical outcomes were broadly comparable to those in South Korea. Both countries had income gradients in health care utilization and spending, with more pronounced differences between the lowest and highest income deciles in the US. These findings underscore the persistence of systemic, income-based health inequalities. Effectively addressing health disparities may require coordinated multisectoral policy interventions.

## Notes

Supplement 1. **eTable 1.** Outcome definitions and data sources **eTable 2**. Sample characteristics **eTable 3.** Unadjusted outcome values **eTable 4.** Differences between countries by income decile: interaction term results **eTable 5.** Income inequalities in healthcare spending, utilization, and access to care among adults aged 40 and older in the United States and South Korea **eTable 6.** Income inequalities in health status, risk factors, and clinical outcomes among adults aged 40 and older in the United States and South Korea **eTable 7.** Income inequalities in healthcare spending, utilization, and access to care among adults aged 18-64 in the United States and South Korea **eTable 8.** Income inequalities in health status, risk factors, and clinical outcomes among adults aged 18-64 in the United States and South Korea **eTable 9.** Income inequalities in healthcare spending, utilization, and access to care among adults aged 65 and older in the United States and South Korea **eTable 10.** Income inequalities in health status, risk factors, and clinical outcomes among adults aged 65 and older in the United States and South Korea **eTable 11.** Income inequalities in healthcare spending, utilization, and access to care among adults in the United States and South Korea after 2014 **eTable 12.** Income inequalities in health status, risk factors, and clinical outcomes among adults in the United States and South Korea after 2014 **eFigure 1.** Comparison of population age and sex distributions in 2019 **eFigure 2.** Trends in health care spending by household income decile **eFigure 3.** Trends in health care utilization by household income decile **eFigure 4.** Trends in access to care by household income decile **eFigure 5.** Trends in behavioral risk factors by household income decile **eFigure 6.** Trends in clinical outcomes by household income decile

Supplement 2.
Data Sharing Statement
