# Error in the Author Affiliations

**DOI:** 10.1001/jamahealthforum.2026.1225

**Published:** 2026-05-01

In the Original Investigation titled “Income-Based Inequalities in Health System Performance in the US and South Korea,” published on March 20, 2026, the incorrect affiliation was listed for the first author Sungchul Park, PhD. The correct affiliation is Department of Health Policy and Management, College of Health Science, Korea University, Seoul, South Korea. This article has been corrected online.^1^
