# Supplementary material for: Income-Based Inequalities in Health System Performance in the US and South Korea
Source: JAMA Health Forum. 2026 Mar 20;7(3):e260136. doi: 10.1001/jamahealthforum.2026.0136 (PMC13005164; doi:10.1001/jamahealthforum.2026.0136)
Supplement: Supplement 1. — eTable 1. Outcome definitions and data sources eTable 2. Sample characteristics eTable 3. Unadjusted outcome values eTable 4. Differences between countries by income decile: interaction term results eTable 5. Income inequalities in healthcare spending, utilization, and access to care among adults aged 40 and older in the United States and South Korea eTable 6. Income inequalities in health status, risk factors, and clinical outcomes among adults aged 40 and older in the United States and South Korea eTable 7. Income inequalities in healthcare spending, utilization, and access to care among adults aged 18-64 in the United States and South Korea eTable 8. Income inequalities in health status, risk factors, and clinical outcomes among adults aged 18-64 in the United States and South Korea eTable 9. Income inequalities in healthcare spending, utilization, and access to care among adults aged 65 and older in the United States and South Korea eTable 10. Income inequalities in health status, risk factors, and clinical outcomes among adults aged 65 and older in the United States and South Korea eTable 11. Income inequalities in healthcare spending, utilization, and access to care among adults in the United States and South Korea after 2014 eTable 12. Income inequalities in health status, risk factors, and clinical outcomes among adults in the United States and South Korea after 2014 eFigure 1. Comparison of population age and sex distributions in 2019 eFigure 2. Trends in health care spending by household income decile eFigure 3. Trends in health care utilization by household income decile eFigure 4. Trends in access to care by household income decile eFigure 5. Trends in behavioral risk factors by household income decile eFigure 6. Trends in clinical outcomes by household income decile [file jamahealthforum-e260136-s001.pdf]

Supplemental Online Content

Park S, Eggleston K, Kyung Y, Cutler DM. Income-based inequalities in health system performance in the US and South Korea. *JAMA Health Forum*. 2026;7(3):e260136. doi:10.1001/jamahealthforum.2026.0136

eTable 1. Outcome definitions and data sources
eTable 2. Sample characteristics
eTable 3. Unadjusted outcome values
eTable 4. Differences between countries by income decile: interaction term results
eTable 5. Income inequalities in healthcare spending, utilization, and access to care among adults aged 40 and older in the United States and South Korea
eTable 6. Income inequalities in health status, risk factors, and clinical outcomes among adults aged 40 and older in the United States and South Korea
eTable 7. Income inequalities in healthcare spending, utilization, and access to care among adults aged 18-64 in the United States and South Korea
eTable 8. Income inequalities in health status, risk factors, and clinical outcomes among adults aged 18-64 in the United States and South Korea
eTable 9. Income inequalities in healthcare spending, utilization, and access to care among adults aged 65 and older in the United States and South Korea
eTable 10. Income inequalities in health status, risk factors, and clinical outcomes among adults aged 65 and older in the United States and South Korea
eTable 11. Income inequalities in healthcare spending, utilization, and access to care among adults in the United States and South Korea after 2014
eTable 12. Income inequalities in health status, risk factors, and clinical outcomes among adults in the United States and South Korea after 2014
eFigure 1. Comparison of population age and sex distributions in 2019
eFigure 2. Trends in health care spending by household income decile
eFigure 3. Trends in health care utilization by household income decile
eFigure 4. Trends in access to care by household income decile
eFigure 5. Trends in behavioral risk factors by household income decile
eFigure 6. Trends in clinical outcomes by household income decile

This supplemental material has been provided by the authors to give readers additional information about their work.

© 2026 Park S et al. *JAMA Health Forum*.

eTable 1. Outcome definitions and data sources

Outcomes	Definition	Data source	
		United States	South Korea
Health care spending			
Total	Annual expenditures on health care services and goods, including insurance-covered and out-of-pocket costs, expressed in 2021 US dollars	MEPS, 2010-2019	Health Panel Study, 2010-2018
Inpatient admissions	Annual expenditures on inpatient admissions, expressed in 2021 US dollars	MEPS, 2010-2019	Health Panel Study, 2010-2018
Outpatient visits	Annual expenditures on outpatient visits, expressed in 2021 US dollars	MEPS, 2010-2019	Health Panel Study, 2010-2018
Emergency department visits	Annual expenditures on emergency department visits, expressed in 2021 US dollars	MEPS, 2010-2019	Health Panel Study, 2010-2018
Health care utilization			
Type of care			
Inpatient admissions	Annual number of inpatient admissions per 1,000 people	MEPS, 2010-2019	Health Panel Study, 2010-2019
Outpatient visits	Annual number of outpatient visits per 1,000 people	MEPS, 2010-2019	Health Panel Study, 2010-2019
Emergency department visits	Annual number of emergency department visits per 1,000 people	MEPS, 2010-2019	Health Panel Study, 2010-2019
Preventive care			
Routine checkup	Had a usual source of care during the year	MEPS, 2010-2016	KNHANES, 2010-2019
Dental checkup	Received dental care during the year	MEPS, 2010-2016	KNHANES, 2010-2019
Flu vaccination	Received a flu vaccine during the year for individuals aged 50 years and older	MEPS, 2010-2016	KNHANES, 2010-2019
Breast cancer screening [a]	Received breast cancer screening for women aged 50 to 75 years	MEPS, 2010-2016	KNHANES, 2010-2019
Colorectal cancer screening [b]	Received colorectal cancer screening for individuals aged 50 to 75 years	MEPS, 2010-2016	KNHANES, 2010-2019
Cervical cancer screening [c]	Received cervical cancer screening for individuals aged 21 to 65 years	MEPS, 2010-2016	KNHANES, 2010-2019
Access to care			
Having a usual source of care	Had a usual source of care during the year	MEPS, 2010-2019	Health Panel Study, 2012-2013 and 2016-2019

© 2026 Park S et al. *JAMA Health Forum*.

Unmet need for medical care [d]	Reported an unmet need for medical care during the year	MEPS, 2010-2017	Health Panel Study, 2011-2019
Unmet need for medical care due to costs [d]	Reported an unmet need for medical care due to financial reasons during the year	MEPS, 2010-2017	Health Panel Study, 2011-2019
Health status			
Self-reported good health	Reported physical health status, with 'excellent' and 'very good' categorized as good, and all other ratings categorized as poor	MEPS, 2010-2019	Health Panel Study, 2011-2019
Behavioral risk factors			
Ever smoke	Reported having smoked at least 100 cigarettes (or an equivalent threshold) in their lifetime, regardless of current smoking status	NHANES, 2009-2018	KNHANES, 2010-2019
Current smoker	Reported having smoked at least 100 cigarettes (or an equivalent threshold) in their lifetime and currently smokes cigarettes	NHANES, 2009-2018	NHANES, 2010-2019
Overweight	Reported BMI between 25 and 30	NHANES, 2009-2018	KNHANES, 2010-2019
Obese	Reported BMI exceeding 30	NHANES, 2009-2018	KNHANES, 2010-2019
Excessive drinking	Reported consuming over 4 drinks in a single day or more than 14 drinks per week for men, and over 3 drinks in a single day or more than 7 drinks per week for women	NHANES, 2009-2018	KNHANES, 2010-2019
Clinical outcomes			
Major depressive disorder	Reported PHQ-9 (Patient Health Questionnaire) depression score exceeding 15	NHANES, 2009-2018	KNHANES, 2014, 2016, 2018
Uncontrolled hypertension (systolic)	Reported systolic blood pressure exceeding 130 mmHg	NHANES, 2009-2018	KNHANES, 2010-2019
Uncontrolled hypertension (diastolic)	Reported diastolic blood pressure exceeding 90 mmHg	NHANES, 2009-2018	KNHANES, 2010-2019
Uncontrolled diabetes mellitus	Reported HemoglobinA1c exceeding 6.5%	NHANES, 2009-2018	KNHANES, 2010-2019
Elevated total cholesterol	Reported total cholesterol exceeding 200 mg/dL	NHANES, 2009-2018	KNHANES, 2010-2019
Low HDL-cholesterol	Reported HDL-cholesterol below 40 mg/dL	NHANES, 2009-2018	KNHANES, 2010-2019
Elevated LDL-cholesterol	Reported LDL-cholesterol exceeding 130 mg/dL	NHANES, 2009-2018	KNHANES, 2010-2019
Hypertriglyceridemia	Reported triglyceride levels exceeding 150 mg/d	NHANES, 2009-2018	KNHANES, 2010-2019

[a] In the United States, breast cancer screening was defined as having undergone a mammogram within the past two years. In contrast, the South Korean data, which did not include detailed information on screening methods, assessed whether individuals had received breast cancer screening within the past year.

© 2026 Park S et al. *JAMA Health Forum*.

[b] In the United States, colorectal cancer screening was defined as having undergone a colonoscopy within the past ten years, a sigmoidoscopy within the past five years, or a fecal occult blood test within the past year. In contrast, the South Korean data, lacking detailed information on screening methods, assessed whether individuals had received any form of colorectal cancer screening within the past year.

[c] In the United States, cervical cancer screening was defined as having undergone a Papanicolaou (Pap) test within the past three years. In contrast, the South Korean data, which did not include detailed screening information, assessed whether individuals had received any cervical cancer screening within the past year.

[d] An unmet need for medical care was defined using a single question in the South Korean data. In the United States, however, this was determined by combining two related questions regarding delayed care and inability to access care.

© 2026 Park S et al. *JAMA Health Forum*.

eTable 2. Sample characteristics

	United States		South Korea	
	MEPS	NHANES	Health Panel Study	NHANES
Sample size, N	197,002	27,166	115,070	64,382
Period, year	2010-2019	2009-2018	2010-2019	2010-2019
Age, mean (SD)	46.6 (18.0)	46.5 (17.4)	47.7 (16.2)	50.5 (17.1)
Sex, %				
Male	48.8	48.2	47.6	43.8
Female	51.1	51.7	52.4	56.1

© 2026 Park S et al. *JAMA Health Forum*.

eTable 3. Unadjusted outcome values

Outcomes	Mean (SD) or %			
	United States		South Korea	
	Lowest income group	Highest income group	Lowest income group	Highest income group
Health care spending, US$				
Total	7666 (18755)	6476 (18022)	1700 (3696)	732 (2371)
Inpatient admissions	2447 (12081)	1456 (12142)	820 (3061)	258 (1777)
Outpatient visits	1477 (4373)	1942 (5088)	841 (1583)	451 (1076)
Emergency department visits	366 (1587)	211 (1276)	18 (115)	10 (78)
Health care utilization				
Type of care, number per 1000 people				
Inpatient admissions	166 (528)	67 (327)	327 (879)	114 (454)
Outpatient visits	7300 (16614)	7245 (11947)	33486 (37857)	11190 (16180)
Emergency department visits	390 (935)	108 (395)	161 (820)	81 (338)
Preventive care, %				
Routine checkup	62.8	73.1	56.0	71.0
Dental checkup	24.1	56.7	69.4	80.6
Flu vaccination	53.6	60.7	74.5	43.1
Breast cancer screening	24.1	56.7	48.2	64.8
Colorectal cancer screening	66.9	82.1	47.0	68.2
Cervical cancer screening	47.2	68.3	89.1	99.3
Access to care, %				
Having a usual source of care	63.7	77.9	34.0	16.3
Unmet need for medical care	8.6	2.4	22.6	10.8
Unmet need for medical care due to costs	5.9	0.4	12.3	0.5
Health status, %				
Self-reported good health	73.0	94.7	63.9	94.4
Risk factors, %				
Ever smoke	51.4	32.2	36.6	36.8
Current smoker	30.3	6.6	16.3	16.5
Overweight	26.7	35.2	30.4	25.8
Obese	33.7	31.6	5.0	4.5
Excessive drinking	17.2	9.6	10.5	13.3
Clinical reports, %				
Major depressive disorder	20.4	9.6	7.5	0.4
Uncontrolled hypertension (systolic)	9.8	6.8	19.3	7.0
Uncontrolled hypertension (diastolic)	29.2	22.7	48.3	20.1
Uncontrolled diabetes mellitus	19.7	25.4	32.3	37.4
Elevated total cholesterol	34.0	37.7	37.9	38.5
Low HDL-cholesterol	23.3	16.4	24.9	16.1
Elevated LDL-cholesterol	24.7	25.3	32.7	30.1
Hypertriglyceridemia	23.1	19.5	32.3	25.6

© 2026 Park S et al. *JAMA Health Forum*.

eTable 4. Differences between countries by income decile: interaction term results

Outcomes	Interaction term between country and income decile, estimate (95% CI)	P value
Health care spending, US$		
Total	695 (680, 711)	0.000
Inpatient admissions	155 (145, 165)	0.000
Outpatient visits	169 (165, 174)	0.000
Emergency department visits	31 (30, 33)	0.000
Health care utilization		
Type of care, number per 1000 people		
Inpatient admissions	-0.01 (-0.01, -0.01)	0.000
Outpatient visits	-0.80 (-0.82, -0.78)	0.000
Emergency department visits	0.01 (0.01, 0.01)	0.000
Preventive care, %		
Routine checkup	0.01 (0.01, 0.01)	0.000
Dental checkup	0.01 (0.01, 0.01)	0.000
Flu vaccination	0.01 (0.01, 0.01)	0.000
Breast cancer screening	0.02 (0.02, 0.02)	0.000
Colorectal cancer screening	-0.01 (-0.01, -0.01)	0.000
Cervical cancer screening	-0.01 (-0.01, -0.01)	0.000
Access to care, %		
Having a usual source of care	0.04 (0.04, 0.04)	0.000
Unmet need for medical care	-0.01 (-0.01, -0.01)	0.000
Unmet need for medical care due to costs	0.00 (0.00, 0.00)	0.000
Health status, %		
Self-reported good health	0.00 (0.00, 0.00)	0.000
Risk factors, %		
Ever smoke	-0.02 (-0.03, -0.02)	0.000
Current smoker	-0.02 (-0.02, -0.01)	0.000
Overweight	0.01 (0.01, 0.01)	0.000
Obese	-0.01 (-0.01, -0.01)	0.000
Excessive drinking	-0.01 (-0.01, -0.01)	0.001
Clinical reports, %		
Major depressive disorder	-0.01 (-0.01, -0.01)	0.000
Uncontrolled hypertension (systolic)	-0.01 (-0.01, -0.01)	0.000
Uncontrolled hypertension (diastolic)	0.00 (-0.01, 0.00)	0.363
Uncontrolled diabetes mellitus	-0.01 (-0.01, -0.01)	0.048
Elevated total cholesterol	0.00 (-0.01, 0.00)	0.074
Low HDL-cholesterol	0.00 (-0.01, 0.00)	0.124
Elevated LDL-cholesterol	0.00 (-0.01, 0.00)	0.468
Hypertriglyceridemia	0.00 (-0.01, 0.00)	0.243

© 2026 Park S et al. *JAMA Health Forum*.

eTable 5. Income inequalities in healthcare spending, utilization, and access to care among adults aged 40 and older in the United States and South Korea

	United States			South Korea		
	Adjusted values, mean or % (95% CI)		Change associated with an upward shift in household income decile [b], estimate (95% CI)	Adjusted values, mean or % (95% CI)		Change associated with an upward shift in household income decile, estimate (95% CI)
Outcomes	Lowest income group	Highest income group		Lowest income group	Highest income group	
Healthcare spending, US$						
Total	11632 (11060 to 12204)	8469 (7970 to 8968)	-277 (-336 to -219)	1229 (1138 to 1320)	1166 (1051 to 1280)	-43 (-52 to -33)
Inpatient admissions	3420 (3083 to 3757)	1957 (1621 to 2292)	-159 (-195 to -123)	529 (465 to 592)	432 (336 to 528)	-30 (-38 to -22)
Outpatient visits	2129 (2001 to 2257)	2502 (2375 to 2629)	48 (33 to 64)	660 (625 to 694)	699 (652 to 746)	-13 (-17 to -8)
Emergency department visits	414 (369 to 458)	224 (195 to 254)	-16 (-21 to -11)	11 (9 to 13)	8 (7 to 10)	0 (-1 to 0)
Healthcare utilization						
Type of care, number per 1000 people						
Inpatient admissions	200 (186 to 214)	85 (76 to 95)	-12.2 (-13.5 to -10.9)	259 (237 to 281)	188 (166 to 210)	-11.2 (-13.4 to -9.1)
Outpatient visits	10115 (9627 to 10604)	9145 (8843 to 9447)	-5.2 (-46.6 to 36.2)	30351 (29357 to 31345)	21184 (20416 to 21952)	-954.6 (-1033.5 to -875.6)
Emergency department visits	408 (385 to 431)	121 (111 to 130)	-27 (-28.8 to -25.3)	120 (105 to 135)	95 (83 to 107)	-3.8 (-5.2 to -2.4)
Preventive care, %						
Routine checkup	73.6 (72.5 to 74.8)	80.9 (80 to 81.8)	1.1 (1 to 1.2)	59.9 (58.1 to 61.8)	81 (79.8 to 82.2)	1.7 (1.5 to 1.9)
Dental checkup	53.1 (51.7 to 54.6)	64.9 (63.6 to 66.2)	1.4 (1.2 to 1.5)	49.3 (47.2 to 51.4)	50.9 (49.1 to 52.7)	-0.1 (-0.3 to 0.1)
Flu vaccination	24.4 (23.4 to 25.5)	62 (60.9 to 63.1)	4.5 (4.4 to 4.6)	72.6 (70.2 to 75.1)	80.5 (78.9 to 82)	0.7 (0.5 to 0.9)
Breast cancer screening	66.6 (64.5 to 68.7)	82.7 (81.1 to 84.4)	2.1 (1.9 to 2.3)	51.8 (47 to 56.5)	69.1 (64.5 to 73.8)	1.4 (0.8 to 2.1)
Colorectal cancer screening	47.7 (46 to 49.3)	70.1 (68.7 to 71.4)	2.6 (2.4 to 2.8)	48.6 (44.8 to 52.5)	71 (67.7 to 74.3)	1.9 (1.5 to 2.4)

© 2026 Park S et al. *JAMA Health Forum*.

Cervical cancer screening	81.7 (80.9 to 82.6)	84.7 (83.4 to 86)	0.2 (0.1 to 0.3)	95.6 (94.8 to 96.4)	96.4 (96.1 to 96.6)	-0.1 (-0.2 to -0.1)
Access to care, %						
Having a usual source of care	78.5 (77.5 to 79.5)	89.1 (88.4 to 89.7)	1.3 (1.2 to 1.4)	26.9 (25.1 to 28.8)	24.3 (22.7 to 25.8)	-0.2 (-0.4 to -0.1)
Unmet need for medical care	11 (10.2 to 11.8)	2.6 (2.2 to 2.9)	-0.8 (-0.9 to -0.8)	27.6 (25.8 to 29.3)	11.1 (10.2 to 12.1)	-1.1 (-1.2 to -1)
Unmet need for medical care due to costs	7.5 (6.8 to 8.2)	0.4 (0.3 to 0.5)	-0.8 (-0.8 to -0.7)	14.5 (12.9 to 16.1)	0.5 (0.3 to 0.8)	-1.1 (-1.2 to -1.1)

© 2026 Park S et al. *JAMA Health Forum*.

eTable 6. Income inequalities in health status, risk factors, and clinical outcomes among adults aged 40 and older in the United States and South Korea

Outcomes	United States			South Korea		
	Adjusted values, mean or % (95% CI)		Change associated with an upward shift in household income decile, estimate (95% CI)	Adjusted values, mean or % (95% CI)		Change associated with an upward shift in household income decile, estimate (95% CI)
	Lowest income group	Highest income group		Lowest income group	Highest income group	
Health status, %						
Self-reported good health	62.6 (61.5 to 63.8)	93.4 (92.9 to 94)	3.2 (3.1 to 3.3)	71.8 (70.3 to 73.2)	89.8 (88.8 to 90.8)	2 (1.9 to 2.1)
Risk factors, %						
Ever smoke	58.9 (56.6 to 61.2)	30.3 (27.8 to 32.8)	-3.3 (-3.6 to -2.9)	46.6 (45.3 to 48)	35.1 (34.2 to 36)	-0.9 (-1.1 to -0.8)
Current smoker	29.1 (27.4 to 30.7)	2.3 (0.6 to 4.1)	-3.3 (-3.6 to -3)	26.1 (24.7 to 27.4)	12.1 (11.1 to 13.1)	-1.3 (-1.5 to -1.2)
Overweight	30.5 (28.2 to 32.8)	39.4 (36.9 to 41.8)	0.8 (0.4 to 1.1)	28.3 (25.1 to 31.5)	29 (26.7 to 31.2)	0 (-0.3 to 0.3)
Obese	38.6 (36.3 to 40.8)	27.1 (24.7 to 29.6)	-1.4 (-1.8 to -1.1)	6.2 (4.6 to 7.9)	2.9 (2 to 3.8)	-0.4 (-0.5 to -0.2)
Excessive drinking	19.8 (17.2 to 22.3)	7.7 (4.3 to 11.1)	-0.4 (-0.9 to 0)	17 (15.4 to 18.7)	12.8 (11.7 to 13.9)	-0.5 (-0.7 to -0.3)
Clinical outcomes, %						
Major depressive disorder	21.8 (19.3 to 24.2)	9 (7.1 to 10.8)	-1.3 (-1.5 to -1)	9.4 (6.7 to 12.1)	0.2 (0 to 0.5)	-0.6 (-0.7 to -0.5)
Uncontrolled hypertension (systolic)	47.4 (44.1 to 50.6)	30.9 (27.5 to 34.2)	-1.5 (-1.9 to -1.1)	19.2 (17.7 to 20.7)	15.1 (14.2 to 16.1)	-0.4 (-0.6 to -0.3)
Uncontrolled hypertension (diastolic)	26.9 (24 to 29.7)	25.1 (21.6 to 28.6)	-0.5 (-0.9 to -0.1)	14.5 (13.3 to 15.7)	13.6 (12.5 to 14.6)	-0.1 (-0.2 to 0.1)
Uncontrolled diabetes mellitus	17.2 (15 to 19.4)	8.6 (6.5 to 10.6)	-1.1 (-1.3 to -0.9)	16.3 (14.8 to 17.9)	11 (10.1 to 11.9)	-0.5 (-0.6 to -0.3)
Elevated total cholesterol	41.6 (38.3 to 44.8)	47.2 (43.5 to 50.9)	0.6 (0.2 to 1)	12.7 (11.4 to 14)	12 (11 to 13)	0 (-0.2 to 0.1)
Low HDL-cholesterol	26.6 (23.8 to 29.5)	14.9 (12.3 to 17.6)	-1.1 (-1.3 to -0.9)	21.5 (20.1 to 22.8)	16 (15.1 to 17)	-0.4 (-0.6 to -0.3)
Elevated LDL-cholesterol	28.8 (24.6 to 33.1)	31.9 (26.7 to 37.1)	0.1 (-0.5 to 0.6)	70 (66.6 to 73.3)	69.4 (66.7 to 72.1)	-0.1 (-0.5 to 0.2)
Hypertriglyceridemia	29.1 (24.8 to 33.4)	24.5 (19.8 to 29.3)	-0.9 (-1.4 to -0.4)	36.9 (35 to 38.7)	28.9 (27.6 to 30.3)	-0.7 (-0.9 to -0.5)

Abbreviation: HDL, high-density lipoprotein; LDL, low-density lipoprotein.

© 2026 Park S et al. *JAMA Health Forum*.

eTable 7. Income inequalities in healthcare spending, utilization, and access to care among adults aged 18–64 in the United States and South Korea

	United States			South Korea		
	Adjusted values, mean or % (95% CI)		Change associated with an upward shift in household income decile [b], estimate (95% CI)	Adjusted values, mean or % (95% CI)		Change associated with an upward shift in household income decile, estimate (95% CI)
Outcomes	Lowest income group	Highest income group		Lowest income group	Highest income group	
Healthcare spending, US$						
Total	6349 (5971 to 6727)	5340 (5068 to 5611)	-118 (-157 to -79)	868 (793 to 944)	671 (612 to 730)	-21 (-28 to -14)
Inpatient admissions	2007 (1787 to 2228)	1191 (1008 to 1374)	-77 (-100 to -53)	373 (318 to 428)	226 (179 to 273)	-18 (-24 to -12)
Outpatient visits	1321 (1236 to 1407)	1644 (1563 to 1726)	38 (28 to 47)	471 (437 to 505)	429 (402 to 455)	-3 (-5 to 0)
Emergency department visits	375 (340 to 409)	207 (183 to 232)	-13 (-17 to -10)	15 (8 to 22)	7 (5 to 8)	0 (0 to 0)
Healthcare utilization						
Type of care, number per 1000 people						
Inpatient admissions	142 (132 to 151)	52 (47 to 57)	-8.4 (-9.2 to -7.6)	205 (186 to 224)	121 (106 to 137)	-5.9 (-7.4 to -4.5)
Outpatient visits	6536 (6214 to 6859)	5979 (5788 to 6171)	-24.6 (-53.5 to 4.3)	17667 (17020 to 18313)	12942 (12467 to 13417)	-335.3 (-386.4 to -284.1)
Emergency department visits	395 (376 to 414)	97 (90 to 104)	-27.9 (-29.2 to -26.5)	116 (103 to 129)	81 (71 to 91)	-3.1 (-4.2 to -2.1)
Preventive care, %						
Routine checkup	57.6 (56.5 to 58.6)	69.2 (68.2 to 70.2)	1.6 (1.5 to 1.8)	51.2 (49.1 to 53.3)	76.2 (74.8 to 77.6)	2.3 (2.1 to 2.5)
Dental checkup	43.5 (41.4 to 45.6)	54.3 (52.6 to 56)	1.5 (1.3 to 1.7)	50.7 (48.3 to 53)	53.4 (51.1 to 55.7)	0.2 (-0.1 to 0.5)
Flu vaccination	23 (22.1 to 23.9)	54.8 (53.9 to 55.8)	3.9 (3.8 to 4)	81 (78.8 to 83.2)	86.6 (85.2 to 87.9)	0.5 (0.3 to 0.7)
Breast cancer screening	65 (62.3 to 67.7)	82 (80.2 to 83.9)	2.3 (2 to 2.5)	51.8 (47 to 56.5)	69.1 (64.5 to 73.8)	1.2 (0.5 to 1.9)
Colorectal cancer screening	47.6 (45 to 50.1)	71 (69 to 72.9)	2.8 (2.6 to 3)	48.6 (44.8 to 52.5)	71 (67.7 to 74.3)	1.7 (1.1 to 2.2)
Cervical cancer screening	87.7 (86.8 to 88.6)	89.2 (88.1 to 90.4)	0.2 (0.2 to 0.2)	95.6 (94.8 to 96.4)	96.4 (96.1 to 96.6)	-0.1 (-0.1 to 0)

© 2026 Park S et al. *JAMA Health Forum*.

Access to care, %						
Having a usual source of care	63.4 (62.4 to 64.3)	80.2 (79.4 to 80.9)	2 (2 to 2.1)	17.7 (16.2 to 19.1)	16.7 (15.4 to 18)	-0.1 (-0.3 to 0)
Unmet need for medical care	10.6 (9.9 to 11.3)	2.3 (2 to 2.6)	-0.8 (-0.9 to -0.8)	19.9 (18.6 to 21.2)	10.7 (9.7 to 11.7)	-0.7 (-0.8 to -0.6)
Unmet need for medical care due to costs	7.6 (7 to 8.2)	0.5 (0.3 to 0.6)	-0.7 (-0.8 to -0.7)	8.7 (7.8 to 9.6)	0.5 (0.2 to 0.7)	-0.7 (-0.7 to -0.6)

© 2026 Park S et al. *JAMA Health Forum*.

eTable 8. Income inequalities in health status, risk factors, and clinical outcomes among adults aged 18–64 in the United States and South Korea

	United States			South Korea		
	Adjusted values, mean or % (95% CI)		Change associated with an upward shift in household income decile, estimate (95% CI)	Adjusted values, mean or % (95% CI)		Change associated with an upward shift in household income decile, estimate (95% CI)
Outcomes	Lowest income group	Highest income group		Lowest income group	Highest income group	
Health status, %						
Self-reported good health	62.6 (61.5 to 63.8)	93.4 (92.9 to 94)	2.4 (2.3 to 2.5)	71.8 (70.3 to 73.2)	89.8 (88.8 to 90.8)	1.1 (1 to 1.2)
Risk factors, %						
Ever smoke	52.2 (49.8 to 54.5)	24.6 (22.1 to 27.2)	-3.3 (-3.6 to -3)	47.3 (45.7 to 48.9)	33.8 (32.7 to 35)	-1.2 (-1.3 to -1.1)
Current smoker	28 (26.3 to 29.7)	1.6 (-0.3 to 3.5)	-3.3 (-3.5 to -3)	29 (27.4 to 30.6)	13.2 (12.1 to 14.3)	-1.4 (-1.6 to -1.3)
Overweight	29.6 (27.3 to 31.9)	36.5 (34 to 39.1)	0.6 (0.3 to 0.9)	27.9 (25.3 to 30.5)	26 (24 to 27.9)	-0.2 (-0.4 to 0.1)
Obese	35.5 (33.2 to 37.9)	26 (23.4 to 28.5)	-1.3 (-1.6 to -1)	7.6 (6.2 to 9.1)	4.1 (3.2 to 5.1)	-0.5 (-0.7 to -0.3)
Excessive drinking	14.1 (11.8 to 16.5)	6 (0 to 12)	-0.5 (-0.8 to -0.2)	18.8 (16.9 to 20.7)	12 (10.9 to 13.2)	-0.4 (-0.6 to -0.3)
Clinical outcomes, %						
Major depressive disorder	19 (16.6 to 21.4)	8.5 (6.5 to 10.4)	-1.2 (-1.4 to -1)	7.9 (5.9 to 9.8)	0.4 (0.1 to 0.7)	-0.4 (-0.5 to -0.3)
Uncontrolled hypertension (systolic)	34.7 (31.8 to 37.5)	21 (17.6 to 24.4)	-1 (-1.2 to -0.7)	14 (12.5 to 15.5)	9.6 (8.7 to 10.4)	-0.4 (-0.5 to -0.3)
Uncontrolled hypertension (diastolic)	20.7 (18.3 to 23.1)	20.1 (16.6 to 23.6)	-0.3 (-0.6 to 0)	11.7 (10.4 to 12.9)	9.3 (8.3 to 10.3)	-0.2 (-0.3 to -0.1)
Uncontrolled diabetes mellitus	12.3 (10.4 to 14.2)	6 (4 to 8)	-0.6 (-0.8 to -0.5)	11.5 (10.1 to 12.9)	7 (6.3 to 7.8)	-0.4 (-0.5 to -0.3)
Elevated total cholesterol	33.6 (30.5 to 36.7)	39.2 (35.3 to 43)	0.1 (-0.2 to 0.4)	11.3 (10 to 12.6)	10.1 (9.1 to 11.1)	-0.1 (-0.2 to 0)
Low HDL-cholesterol	24.8 (21.9 to 27.7)	15.7 (12.9 to 18.6)	-1.1 (-1.3 to -0.9)	21.5 (20.1 to 22.8)	16 (15.1 to 17)	-0.4 (-0.6 to -0.3)
Elevated LDL-cholesterol	24.3 (20.2 to 28.4)	25.7 (20.5 to 30.8)	-0.2 (-0.6 to 0.2)	69.5 (65.9 to 73)	66.6 (63.6 to 69.5)	0 (-0.3 to 0.3)
Hypertriglyceridemia	23.7 (19.6 to 27.8)	18.4 (13.7 to 23.1)	-0.9 (-1.3 to -0.5)	33.9 (32 to 35.8)	25.2 (23.8 to 26.6)	-0.8 (-1 to -0.6)

Abbreviation: HDL, high-density lipoprotein; LDL, low-density lipoprotein.

© 2026 Park S et al. *JAMA Health Forum*.

eTable 9. Income inequalities in healthcare spending, utilization, and access to care among adults aged 65 and older in the United States and South Korea

	United States			South Korea		
	Adjusted values, mean or % (95% CI)		Change associated with an upward shift in household income decile [b], estimate (95% CI)	Adjusted values, mean or % (95% CI)		Change associated with an upward shift in household income decile, estimate (95% CI)
Outcomes	Lowest income group	Highest income group		Lowest income group	Highest income group	
Healthcare spending, US$						
Total	14170 (13198 to 15141)	12507 (11561 to 13454)	-289 (-401 to -177)	1511 (1374 to 1647)	1527 (1342 to 1711)	-4 (-20 to 12)
Inpatient admissions	4523 (3866 to 5179)	3024 (2499 to 3548)	-195 (-264 to -126)	636 (531 to 741)	607 (456 to 758)	0 (-14 to 14)
Outpatient visits	2556 (2327 to 2785)	3560 (3225 to 3894)	93 (59 to 128)	840 (774 to 906)	883 (794 to 972)	-4 (-10 to 3)
Emergency department visits	336 (281 to 390)	265 (209 to 321)	-8 (-15 to -2)	11 (8 to 14)	18 (13 to 23)	0 (0 to 1)
Healthcare utilization						
Type of care, number per 1000 people						
Inpatient admissions	265 (236 to 294)	171 (146 to 196)	-12.1 (-15.2 to -9)	295 (262 to 327)	249 (200 to 298)	-7.3 (-11.2 to -3.3)
Outpatient visits	11477 (10710 to 12244)	14663 (13955 to 15371)	301.2 (220.6 to 381.8)	39877 (38090 to 41664)	31863 (30309 to 33418)	-938.9 (-1084.6 to -793.1)
Emergency department visits	328 (296 to 361)	198 (174 to 222)	-17.1 (-20.6 to -13.7)	128 (104 to 153)	137 (110 to 164)	0.9 (-1.9 to 3.6)
Preventive care, %						
Routine checkup	88.9 (87.4 to 90.4)	90 (88.7 to 91.3)	0.3 (0.2 to 0.5)	62.4 (59.6 to 65.1)	72.6 (70.3 to 74.9)	0.7 (0.4 to 1.1)
Dental checkup	65.2 (62.8 to 67.5)	78.8 (77 to 80.6)	1.2 (0.9 to 1.4)	78.5 (75.9 to 81.2)	79.1 (76.9 to 81.3)	0.1 (-0.2 to 0.4)
Flu vaccination	28.2 (26.1 to 30.2)	66.7 (64.8 to 68.7)	4.4 (4.2 to 4.6)	72.8 (69 to 76.5)	78.4 (75.7 to 81.1)	0.8 (0.4 to 1.2)
Breast cancer screening	71.2 (67.3 to 75)	84.3 (81.5 to 87.1)	1.7 (1.3 to 2.1)	48.6 (41.1 to 56.1)	61.3 (53.7 to 69)	1.4 (0.4 to 2.4)
Colorectal cancer screening	59.5 (56.3 to 62.7)	79.1 (77 to 81.2)	2.1 (1.8 to 2.4)	50.1 (43.9 to 56.4)	63.9 (58.3 to 69.5)	2 (1.2 to 2.7)

© 2026 Park S et al. *JAMA Health Forum*.

Cervical cancer screening	43.9 (41 to 46.9)	50.5 (47.4 to 53.7)	0.6 (0.4 to 0.8)	86.9 (86.9 to 86.9)	86.9 (86.9 to 86.9)	0 (0 to 0)
Access to care, %						
Having a usual source of care	90.5 (89.2 to 91.8)	95.3 (94.5 to 96.1)	0.4 (0.3 to 0.5)	37.4 (34.3 to 40.6)	34.8 (31.9 to 37.7)	-0.1 (-0.4 to 0.2)
Unmet need for medical care	4.4 (3.4 to 5.4)	3.6 (2.9 to 4.4)	-0.3 (-0.4 to -0.2)	26 (23.8 to 28.2)	9.3 (7.7 to 10.8)	-1.6 (-1.8 to -1.5)
Unmet need for medical care due to costs	1.9 (1.2 to 2.6)	0.3 (0.1 to 0.6)	-0.3 (-0.3 to -0.2)	14.7 (12.9 to 16.5)	2.5 (1.6 to 3.4)	-1.2 (-1.4 to -1.1)

© 2026 Park S et al. *JAMA Health Forum*.

eTable 10. Income inequalities in health status, risk factors, and clinical outcomes among adults aged 65 and older in the United States and South Korea

	United States			South Korea		
	Adjusted values, mean or % (95% CI)		Change associated with an upward shift in household income decile, estimate (95% CI)	Adjusted values, mean or % (95% CI)		Change associated with an upward shift in household income decile, estimate (95% CI)
Outcomes	Lowest income group	Highest income group		Lowest income group	Highest income group	
Health status, %						
Self-reported good health	68.7 (66.7 to 70.6)	89.6 (88.4 to 90.9)	2.4 (2.2 to 2.6)	64.1 (62 to 66.3)	77.8 (75.7 to 80)	1.9 (1.7 to 2.1)
Risk factors, %						
Ever smoke	53 (49.2 to 56.8)	43.4 (39.3 to 47.5)	-1.3 (-1.9 to -0.6)	39 (37.1 to 40.9)	35.1 (33.5 to 36.6)	-0.5 (-0.7 to -0.3)
Current smoker	13.6 (11.5 to 15.8)	4 (1.7 to 6.4)	-1.3 (-1.7 to -1)	13.6 (11.8 to 15.3)	6.7 (5.3 to 8)	-0.7 (-0.9 to -0.5)
Overweight	33.3 (29.4 to 37.2)	38.2 (34.1 to 42.4)	0.5 (-0.1 to 1.1)	26.6 (21.4 to 31.8)	31.5 (27.4 to 35.6)	0.1 (-0.4 to 0.7)
Obese	31.5 (27.8 to 35.1)	26.2 (22.3 to 30.1)	-0.8 (-1.4 to -0.3)	3.6 (1.4 to 5.7)	4.5 (2.7 to 6.3)	-0.1 (-0.3 to 0.2)
Excessive drinking	16.4 (11.6 to 21.2)	5.7 (-1.2 to 12.7)	0.2 (-0.6 to 0.9)	9.6 (7.3 to 12)	6.1 (4.4 to 7.8)	-0.4 (-0.6 to -0.2)
Clinical outcomes, %						
Major depressive disorder	18.8 (15.1 to 22.4)	12.2 (8.9 to 15.5)	-0.9 (-1.2 to -0.5)	9.2 (5.6 to 12.9)	1.6 (0.4 to 2.9)	-0.7 (-1 to -0.5)
Uncontrolled hypertension (systolic)	62.9 (57.5 to 68.4)	46.4 (39.8 to 53)	-1.4 (-2.1 to -0.7)	30.8 (28.3 to 33.3)	30.5 (28.1 to 32.8)	0.2 (-0.1 to 0.5)
Uncontrolled hypertension (diastolic)	17.8 (13.9 to 21.7)	11.4 (6.5 to 16.4)	-0.4 (-0.9 to 0.1)	9.3 (7.7 to 10.8)	7.6 (6.2 to 9)	0 (-0.2 to 0.2)
Uncontrolled diabetes mellitus	23.9 (19.9 to 27.9)	14.2 (9.6 to 18.7)	-1.3 (-1.7 to -0.8)	21.7 (19 to 24.3)	19.8 (17.6 to 21.9)	-0.1 (-0.4 to 0.2)
Elevated total cholesterol	38.5 (33.3 to 43.8)	40.6 (34.5 to 46.7)	0.5 (-0.1 to 1.1)	11.1 (9.2 to 13)	9.6 (7.9 to 11.2)	-0.1 (-0.3 to 0.1)
Low HDL-cholesterol	21 (16.8 to 25.2)	14.6 (10.3 to 18.9)	-1.1 (-1.3 to -0.9)	21.5 (20.1 to 22.8)	16 (15.1 to 17)	-0.4 (-0.6 to -0.3)
Elevated LDL-cholesterol	23.7 (17.2 to 30.2)	26.9 (18.4 to 35.4)	0.1 (-0.8 to 0.9)	62.3 (55.9 to 68.7)	56.5 (50.8 to 62.3)	-0.9 (-1.6 to -0.2)
Hypertriglyceridemia	28.6 (21.8 to 35.4)	14.2 (7.3 to 21.1)	-1.2 (-2 to -0.4)	31.9 (29 to 34.7)	29.7 (27.2 to 32.1)	-0.1 (-0.4 to 0.2)

Abbreviation: HDL, high-density lipoprotein; LDL, low-density lipoprotein.

© 2026 Park S et al. *JAMA Health Forum*.

eTable 11. Income inequalities in healthcare spending, utilization, and access to care among adults in the United States and South Korea after 2014

Outcomes	United States			South Korea		
	Adjusted values, mean or % (95% CI)		Change associated with an upward shift in household income decile, estimate (95% CI)	Adjusted values, mean or % (95% CI)		Change associated with an upward shift in household income decile, estimate (95% CI)
	Lowest income group	Highest income group		Lowest income group	Highest income group	
Healthcare spending, US$						
Total	8271 (7656 to 8885)	6719 (6305 to 7132)	-178 (-231 to -125)	1239 (1140 to 1337)	1098 (989 to 1207)	-38 (-48 to -27)
Inpatient admissions	2474 (2161 to 2786)	1325 (1102 to 1547)	-109 (-139 to -79)	531 (458 to 603)	411 (317 to 504)	-27 (-35 to -18)
Outpatient visits	1551 (1435 to 1667)	2043 (1914 to 2171)	51 (37 to 65)	658 (620 to 695)	669 (621 to 717)	-11 (-15 to -6)
Emergency department visits	385 (337 to 433)	225 (196 to 255)	-13 (-18 to -8)	14 (10 to 18)	12 (9 to 14)	0 (0 to 0)
Healthcare utilization						
Type of care, number per 1000 people						
Inpatient admissions	163 (150 to 176)	62 (55 to 70)	-9.7 (-10.9 to -8.5)	243 (219 to 266)	162 (141 to 184)	-9.4 (-11.4 to -7.3)
Outpatient visits	8358 (7836 to 8880)	7902 (7610 to 8194)	12.5 (-30.3 to 55.4)	25672 (24547 to 26798)	18090 (17310 to 18870)	-735.4 (-812.1 to -658.7)
Emergency department visits	402 (377 to 426)	114 (104 to 124)	-27.2 (-29.1 to -25.4)	121 (102 to 139)	107 (93 to 122)	-3.1 (-4.6 to -1.7)
Preventive care, %						
Routine checkup	64.9 (63.5 to 66.3)	74.2 (73 to 75.5)	1.3 (1.1 to 1.4)	51.2 (49.1 to 53.3)	76.2 (74.8 to 77.6)	2.2 (2 to 2.4)
Dental checkup	53.9 (51.5 to 56.2)	65.2 (63.3 to 67.2)	1.2 (1 to 1.5)	50.7 (48.3 to 53)	53.4 (51.1 to 55.7)	0.1 (-0.2 to 0.4)
Flu vaccination	24.2 (23 to 25.3)	57.4 (56.3 to 58.6)	4 (3.9 to 4.2)	81 (78.8 to 83.2)	86.6 (85.2 to 87.9)	0.6 (0.4 to 0.8)
Breast cancer screening	68.6 (65.4 to 71.8)	81.9 (79.5 to 84.3)	1.7 (1.3 to 2)	51.8 (47 to 56.5)	69.1 (64.5 to 73.8)	1.4 (0.8 to 2.1)
Colorectal cancer screening	47.6 (45 to 50.1)	71 (69 to 72.9)	2.6 (2.4 to 2.9)	48.6 (44.8 to 52.5)	71 (67.7 to 74.3)	1.9 (1.5 to 2.4)

© 2026 Park S et al. *JAMA Health Forum*.

Cervical cancer screening	87.7 (86.8 to 88.6)	89.2 (88.1 to 90.4)	0.2 (0.1 to 0.2)	95.6 (94.8 to 96.4)	96.4 (96.1 to 96.6)	-0.1 (-0.1 to 0)
Access to care, %						
Having a usual source of care	68.6 (67.4 to 69.8)	82.3 (81.4 to 83.2)	1.6 (1.5 to 1.7)	21 (19.3 to 22.7)	21.3 (19.7 to 22.9)	-0.2 (-0.3 to 0)
Unmet need for medical care	8.1 (7.3 to 8.9)	2.6 (2.2 to 3.1)	-0.5 (-0.6 to -0.5)	24.1 (22.2 to 26)	10.1 (9.1 to 11.1)	-0.7 (-0.8 to -0.6)
Unmet need for medical care due to costs	5.2 (4.5 to 5.9)	0.4 (0.3 to 0.6)	-0.5 (-0.5 to -0.4)	11.3 (9.7 to 12.9)	0.4 (0.2 to 0.6)	-0.7 (-0.8 to -0.7)

© 2026 Park S et al. *JAMA Health Forum*.

eTable 12. Income inequalities in health status, risk factors, and clinical outcomes among adults in the United States and South Korea after 2014

	United States			South Korea		
	Adjusted values, mean or % (95% CI)		Change associated with an upward shift in household income decile, estimate (95% CI)	Adjusted values, mean or % (95% CI)		Change associated with an upward shift in household income decile, estimate (95% CI)
Outcomes	Lowest income group	Highest income group		Lowest income group	Highest income group	
Health status, %						
Self-reported good health	62.6 (61.5 to 63.8)	93.4 (92.9 to 94)	2.4 (2.3 to 2.5)	71.8 (70.3 to 73.2)	89.8 (88.8 to 90.8)	1.6 (1.4 to 1.7)
Risk factors, %						
Ever smoke	52.2 (49.8 to 54.5)	24.6 (22.1 to 27.2)	-3 (-3.3 to -2.6)	47.3 (45.7 to 48.9)	33.8 (32.7 to 35)	-1.1 (-1.3 to -0.9)
Current smoker	28 (26.3 to 29.7)	1.6 (-0.3 to 3.5)	-3 (-3.2 to -2.7)	29 (27.4 to 30.6)	13.2 (12.1 to 14.3)	-1.3 (-1.5 to -1.2)
Overweight	29.6 (27.3 to 31.9)	36.5 (34 to 39.1)	0.6 (0.3 to 1)	27.9 (25.3 to 30.5)	26 (24 to 27.9)	-0.1 (-0.3 to 0.2)
Obese	35.5 (33.2 to 37.9)	26 (23.4 to 28.5)	-1 (-1.4 to -0.7)	7.6 (6.2 to 9.1)	4.1 (3.2 to 5.1)	-0.4 (-0.6 to -0.3)
Excessive drinking	14.1 (11.8 to 16.5)	6 (0 to 12)	-0.4 (-0.8 to 0)	18.8 (16.9 to 20.7)	12 (10.9 to 13.2)	-0.6 (-0.7 to -0.4)
Clinical outcomes, %						
Major depressive disorder	19 (16.6 to 21.4)	8.5 (6.5 to 10.4)	-0.9 (-1.2 to -0.7)	7.9 (5.9 to 9.8)	0.4 (0.1 to 0.7)	-0.5 (-0.6 to -0.4)
Uncontrolled hypertension (systolic)	34.7 (31.8 to 37.5)	21 (17.6 to 24.4)	-1.2 (-1.6 to -0.9)	14 (12.5 to 15.5)	9.6 (8.7 to 10.4)	-0.4 (-0.6 to -0.3)
Uncontrolled hypertension (diastolic)	20.7 (18.3 to 23.1)	20.1 (16.6 to 23.6)	-0.2 (-0.5 to 0.1)	11.7 (10.4 to 12.9)	9.3 (8.3 to 10.3)	-0.2 (-0.3 to -0.1)
Uncontrolled diabetes mellitus	12.3 (10.4 to 14.2)	6 (4 to 8)	-0.7 (-0.9 to -0.5)	11.5 (10.1 to 12.9)	7 (6.3 to 7.8)	-0.4 (-0.6 to -0.3)
Elevated total cholesterol	33.6 (30.5 to 36.7)	39.2 (35.3 to 43)	0.4 (0 to 0.8)	11.3 (10 to 12.6)	10.1 (9.1 to 11.1)	-0.1 (-0.2 to 0.1)
Low HDL-cholesterol	24.8 (21.9 to 27.7)	15.7 (12.9 to 18.6)	-1.1 (-1.3 to -0.9)	21.5 (20.1 to 22.8)	16 (15.1 to 17)	-0.4 (-0.6 to -0.3)
Elevated LDL-cholesterol	24.3 (20.2 to 28.4)	25.7 (20.5 to 30.8)	0.2 (-0.4 to 0.7)	69.5 (65.9 to 73)	66.6 (63.6 to 69.5)	-0.4 (-0.8 to 0)
Hypertriglyceridemia	23.7 (19.6 to 27.8)	18.4 (13.7 to 23.1)	-0.7 (-1.1 to -0.2)	33.9 (32 to 35.8)	25.2 (23.8 to 26.6)	-0.8 (-1 to -0.6)

Abbreviation: HDL, high-density lipoprotein; LDL, low-density lipoprotein.

© 2026 Park S et al. *JAMA Health Forum*.

eFigure 1. Comparison of population age and sex distributions in 2019

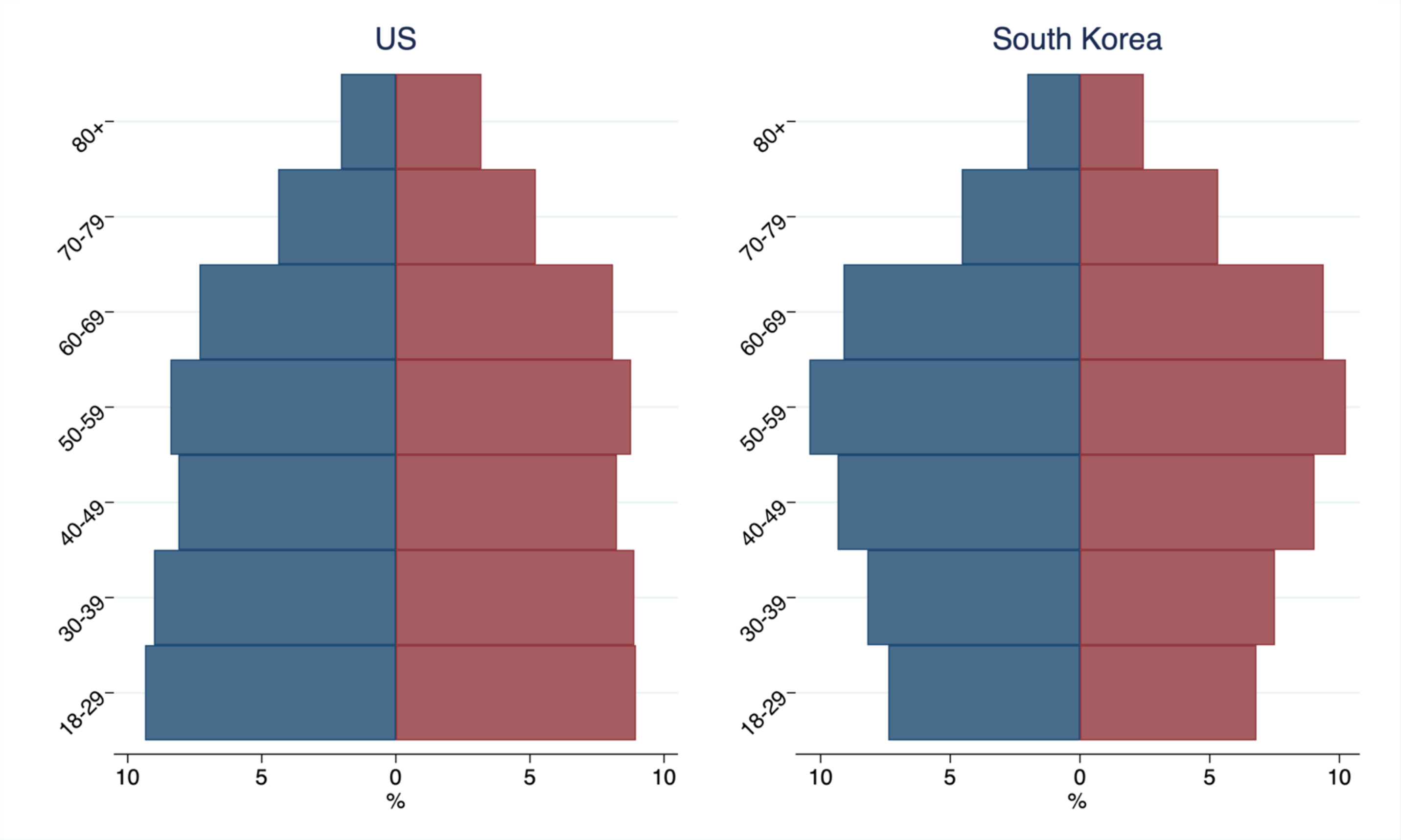

Notes: Data was obtained from the U.S. Census Bureau for the United States and from Statistics Korea for South Korea. The blue represents males and the red females.

© 2026 Park S et al. *JAMA Health Forum*.

eFigure 2. Trends in health care spending by household income decile

Panel A. Spending for inpatient admissions

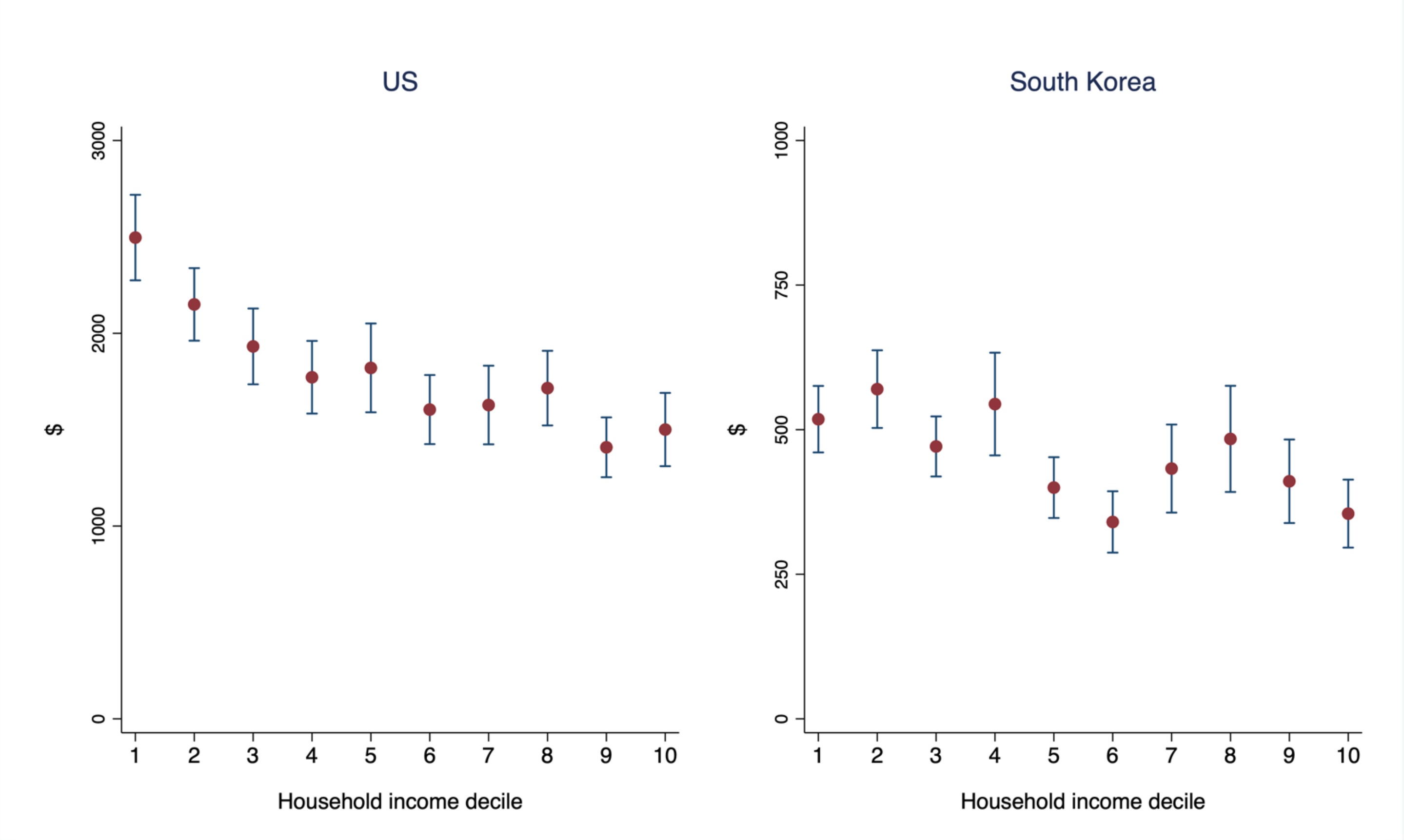

© 2026 Park S et al. *JAMA Health Forum*.

Panel B. Spending for outpatient visits

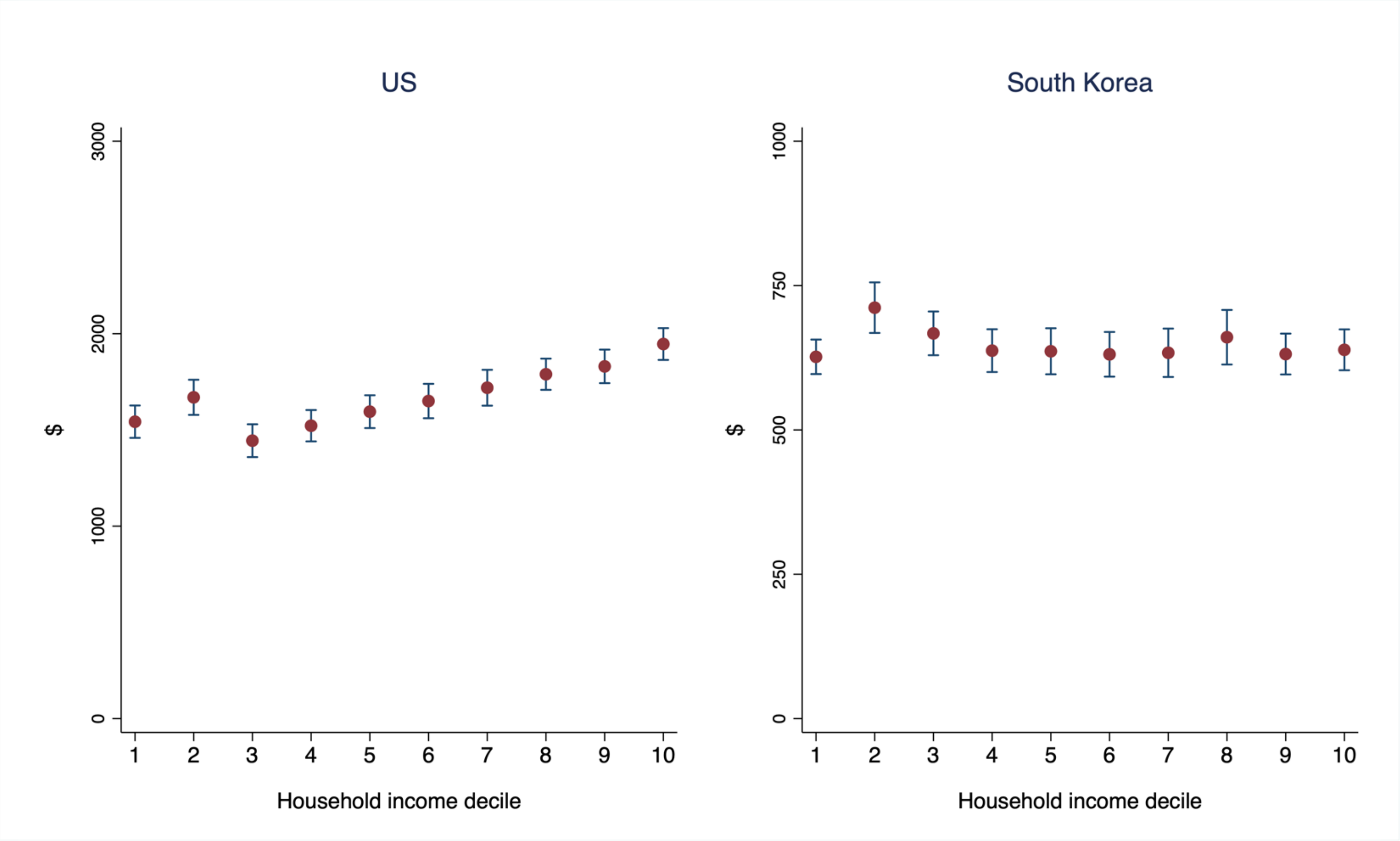

© 2026 Park S et al. *JAMA Health Forum*.

Panel C. Spending for emergency department visits

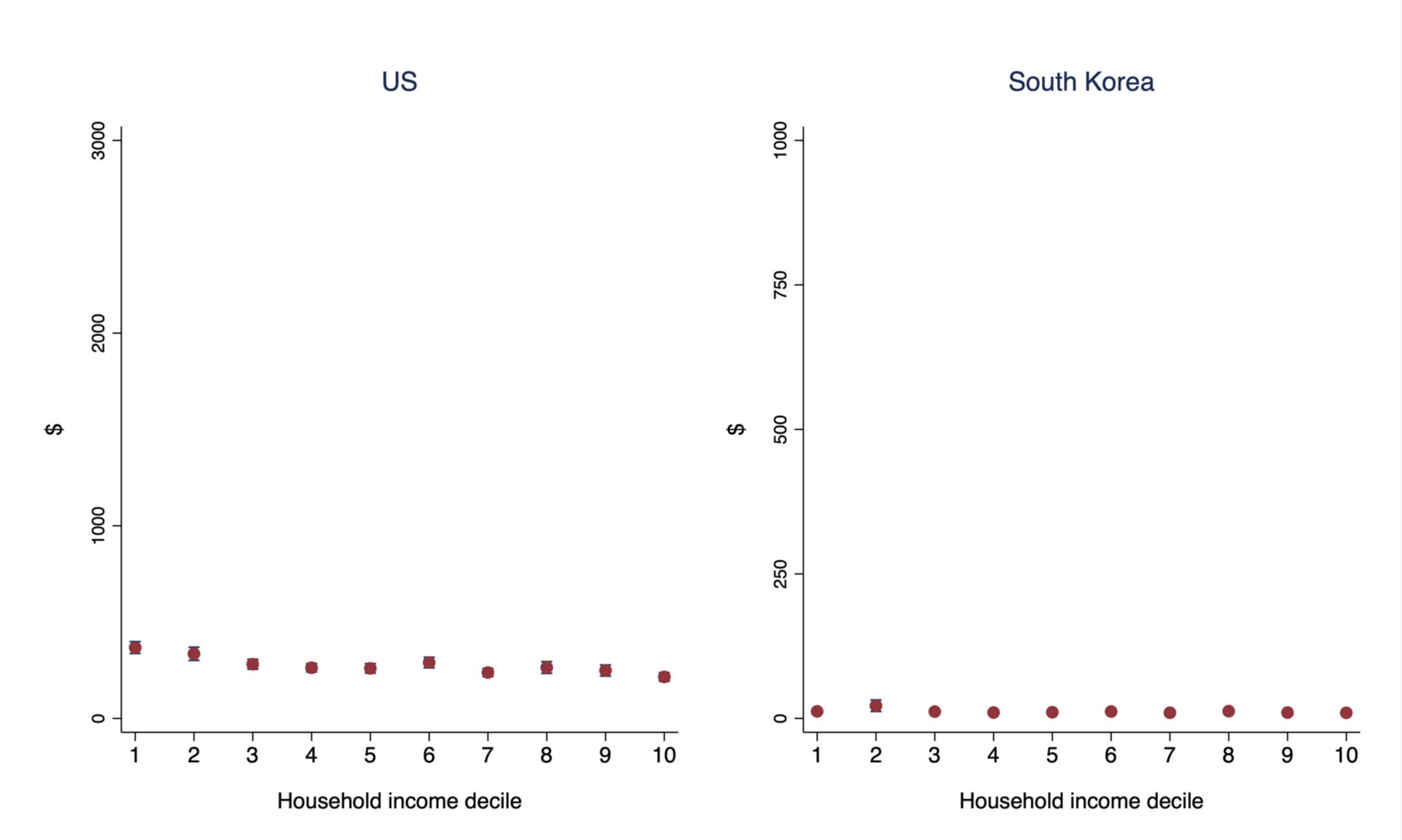

© 2026 Park S et al. *JAMA Health Forum*.

eFigure 3. Trends in health care utilization by household income decile

Panel A. Inpatient admissions

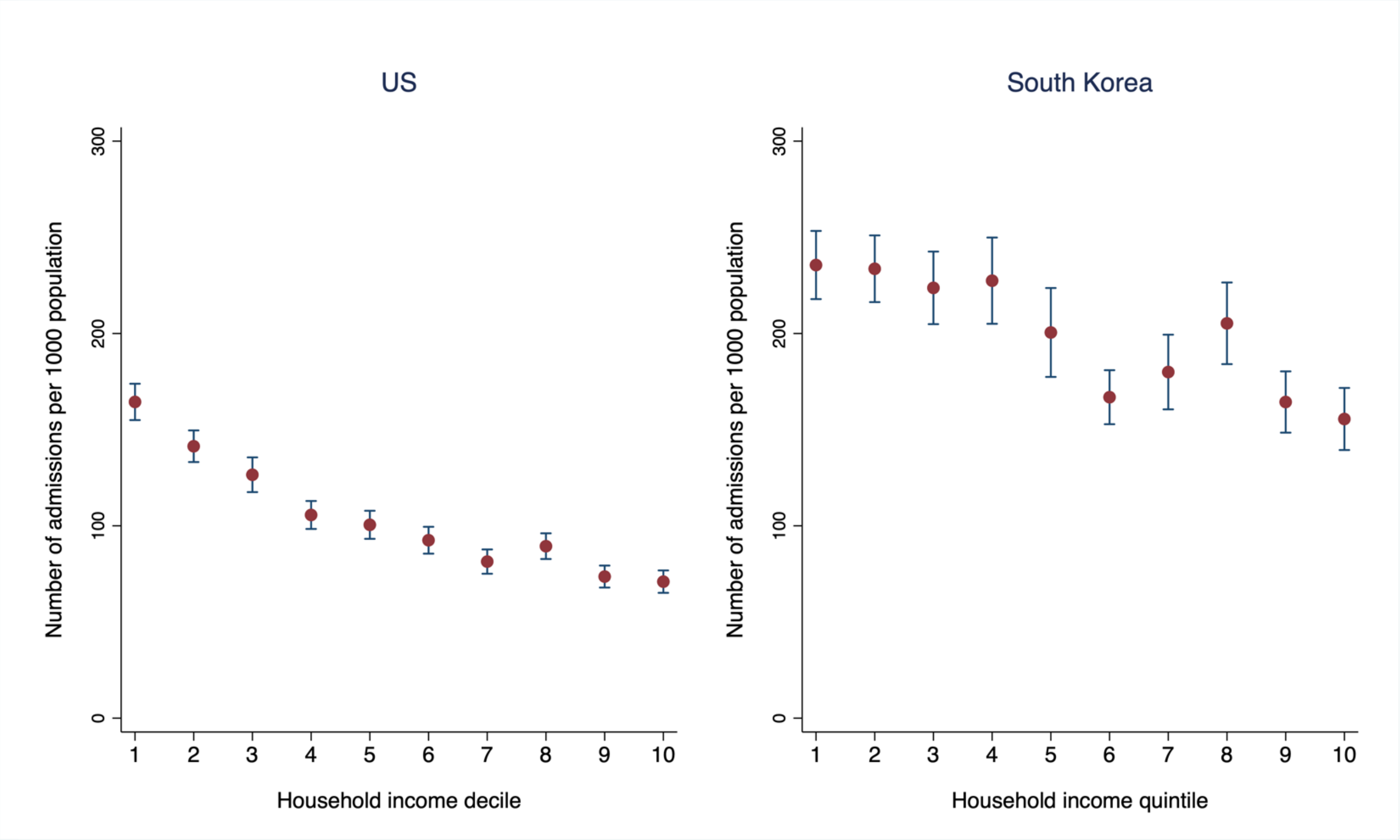

© 2026 Park S et al. *JAMA Health Forum*.

Panel B. Outpatient visits

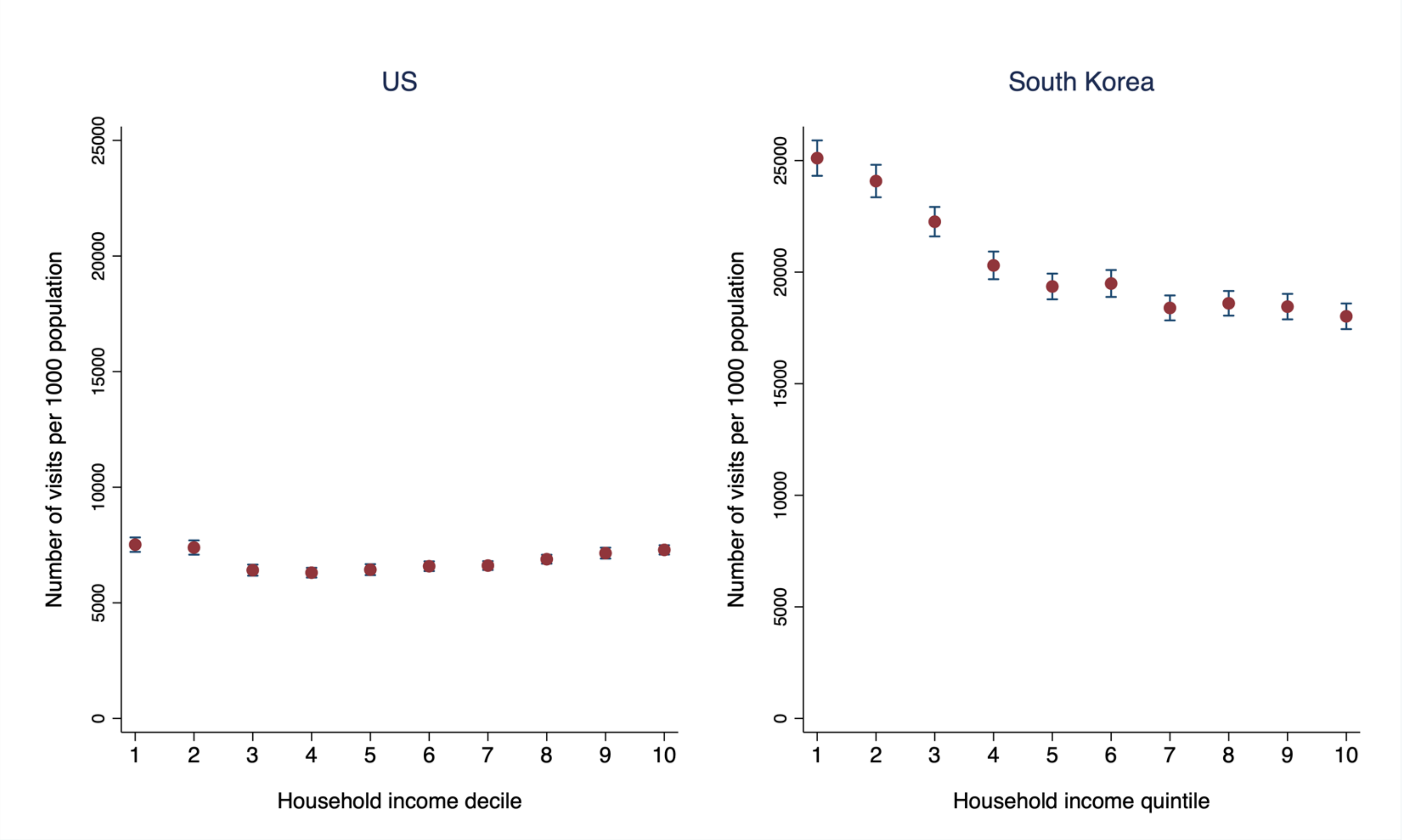

© 2026 Park S et al. *JAMA Health Forum*.

Panel C. Emergency department visits

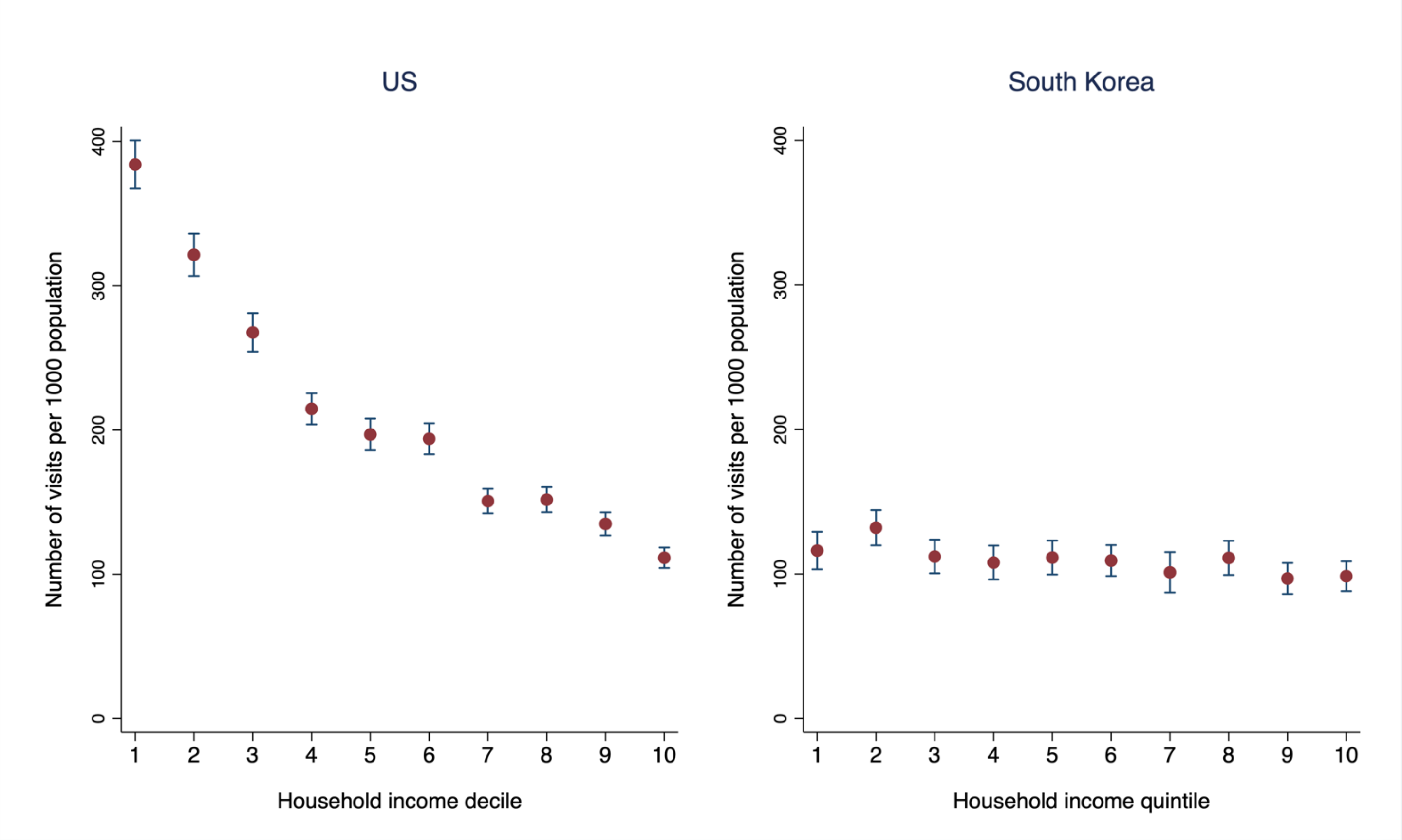

© 2026 Park S et al. *JAMA Health Forum*.

Panel D. Dental checkup

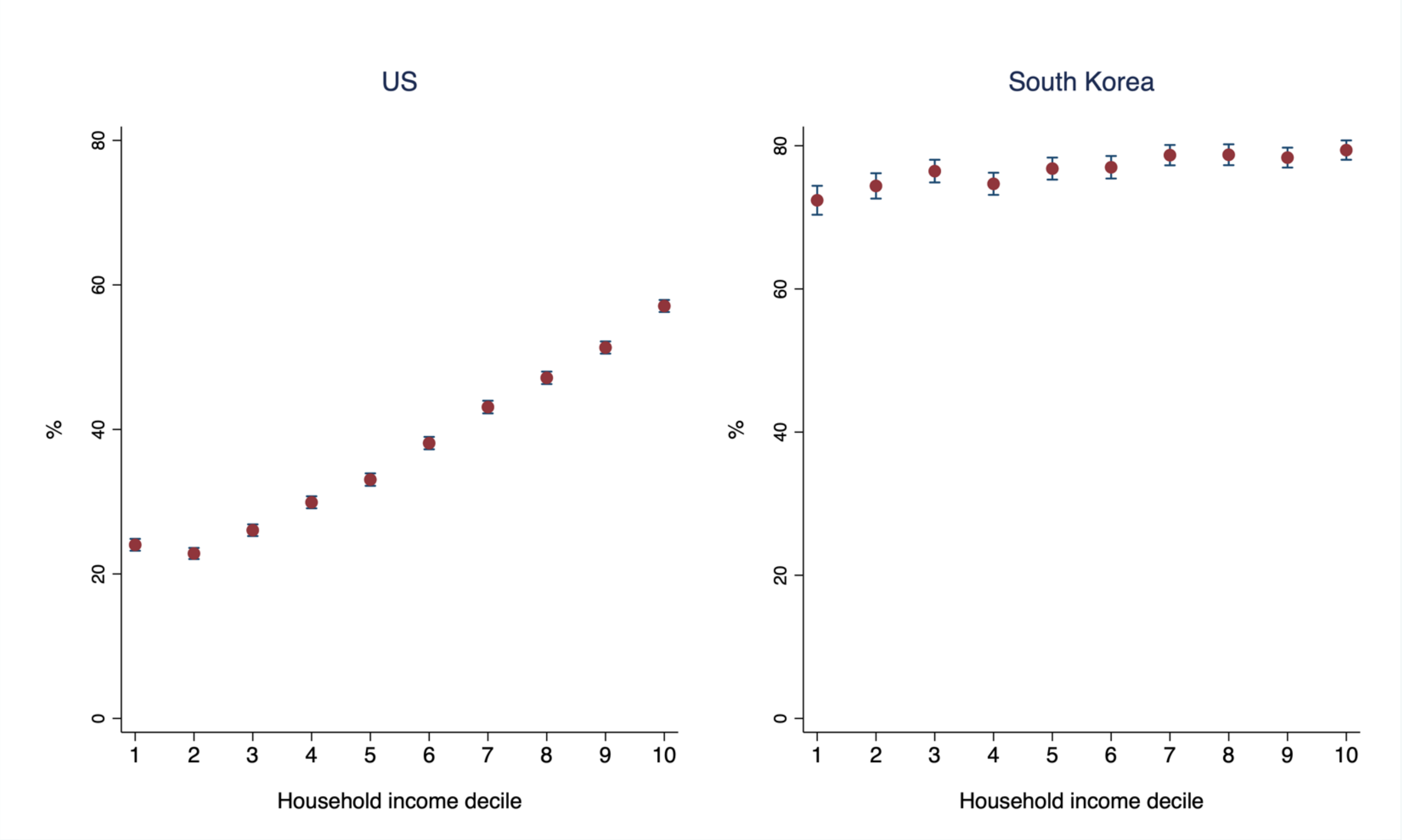

© 2026 Park S et al. *JAMA Health Forum*.

Panel E. Flu vaccination

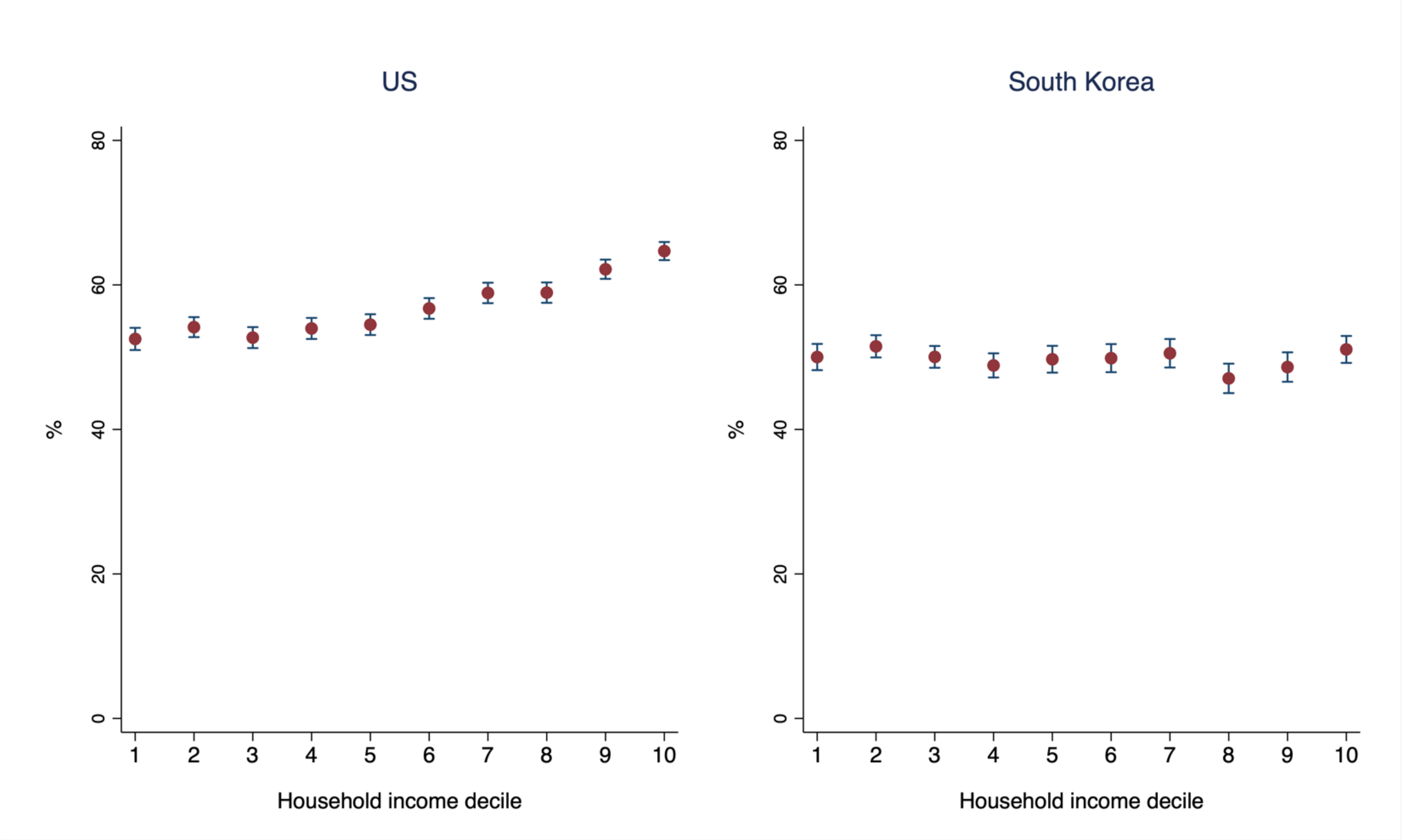

© 2026 Park S et al. *JAMA Health Forum*.

Panel F. Breast cancer screening

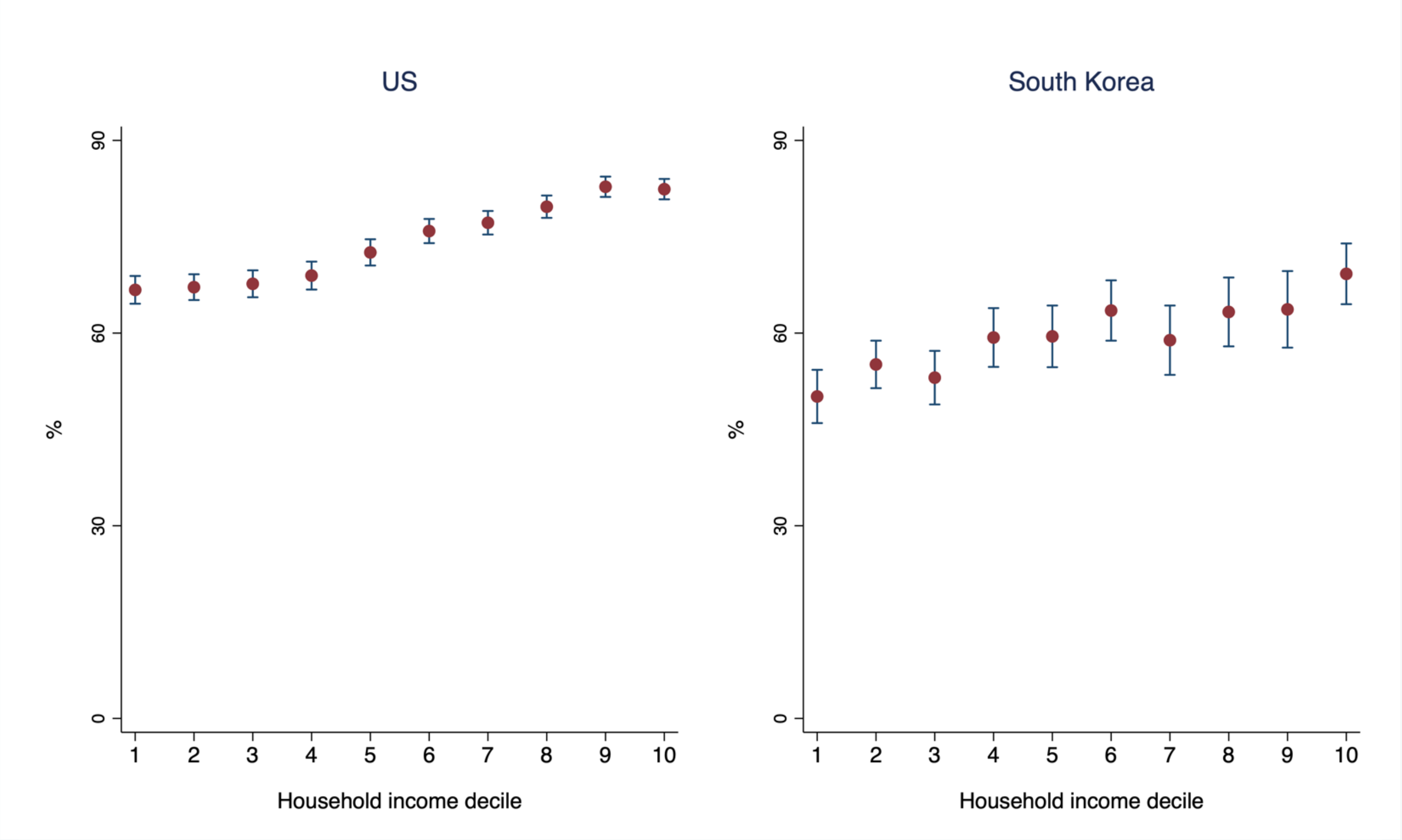

© 2026 Park S et al. *JAMA Health Forum*.

Panel G. Colorectal cancer screening

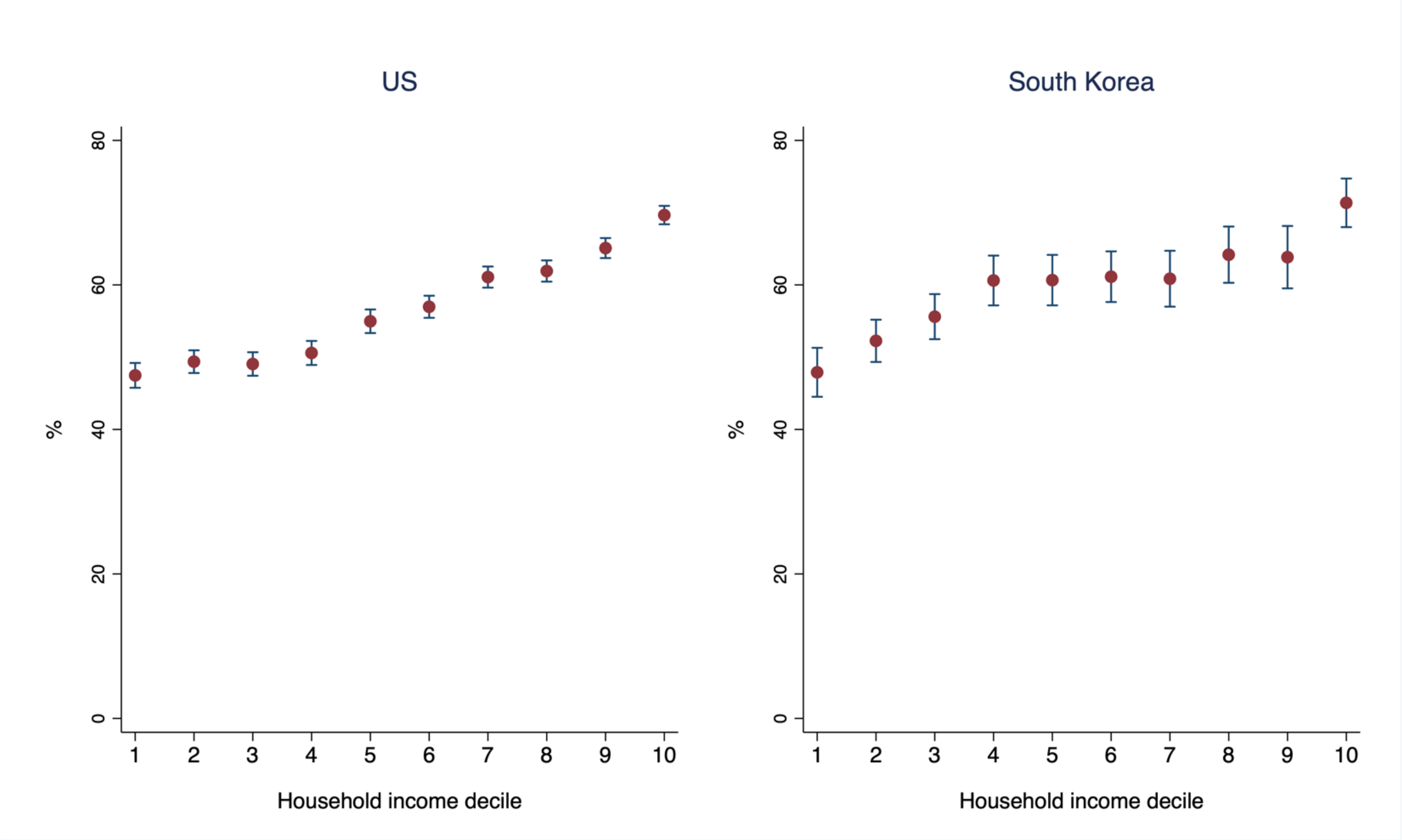

© 2026 Park S et al. *JAMA Health Forum*.

Panel H. Cervical cancer screening

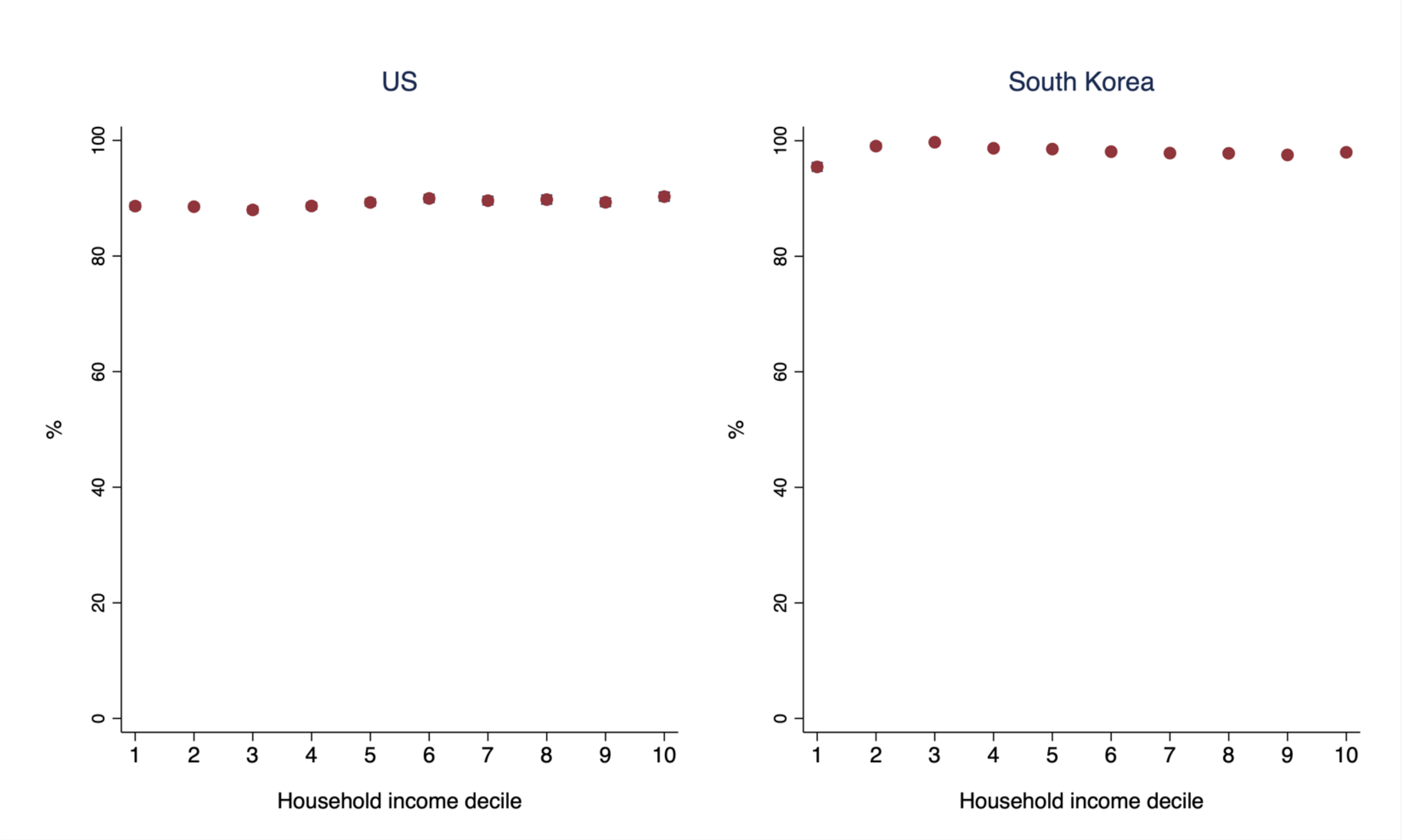

© 2026 Park S et al. *JAMA Health Forum*.

eFigure 4. Trends in access to care by household income decile

Panel A. Unmet need for medical care due to costs

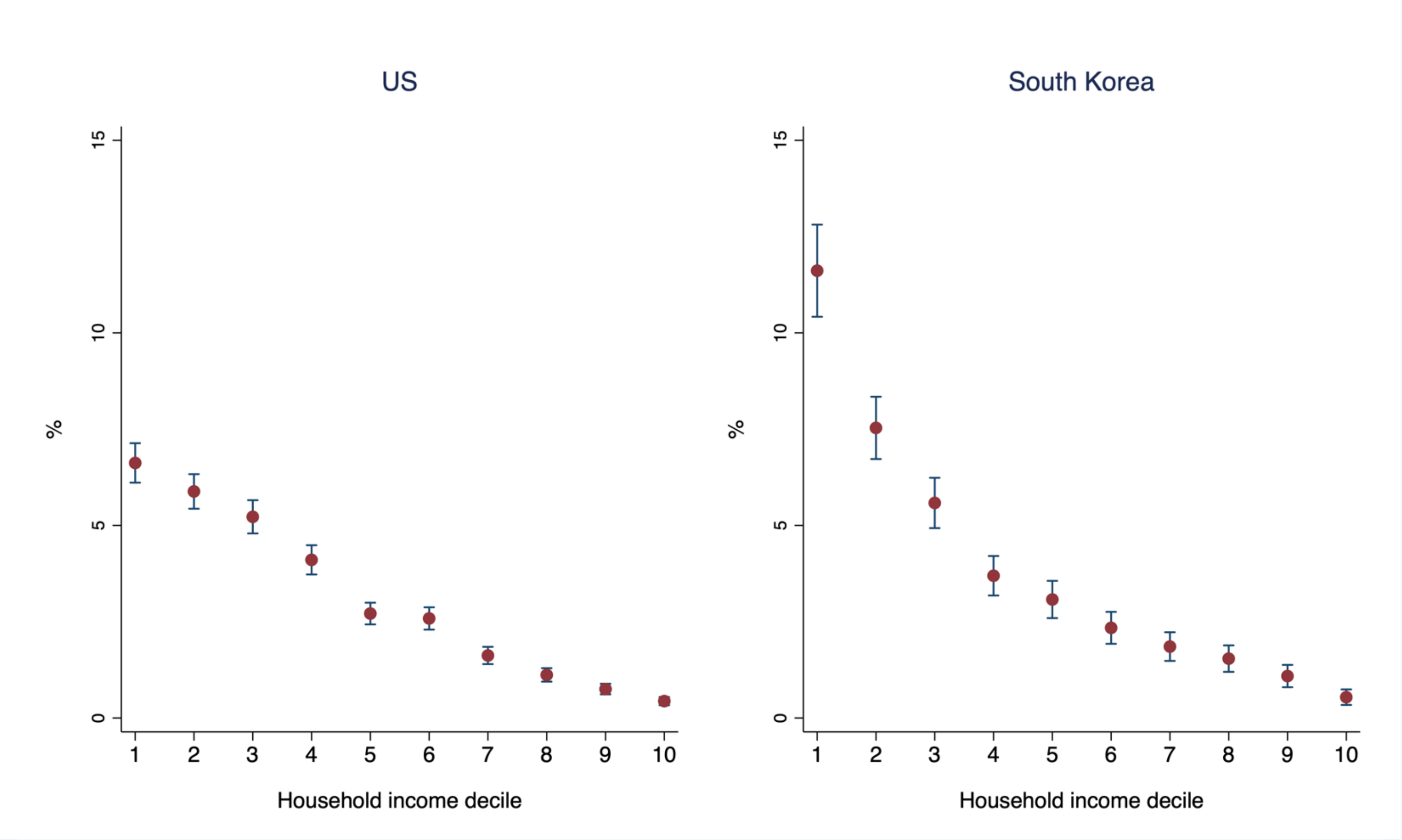

© 2026 Park S et al. *JAMA Health Forum*.

eFigure 5. Trends in behavioral risk factors by household income decile

Panel A. Ever smoke

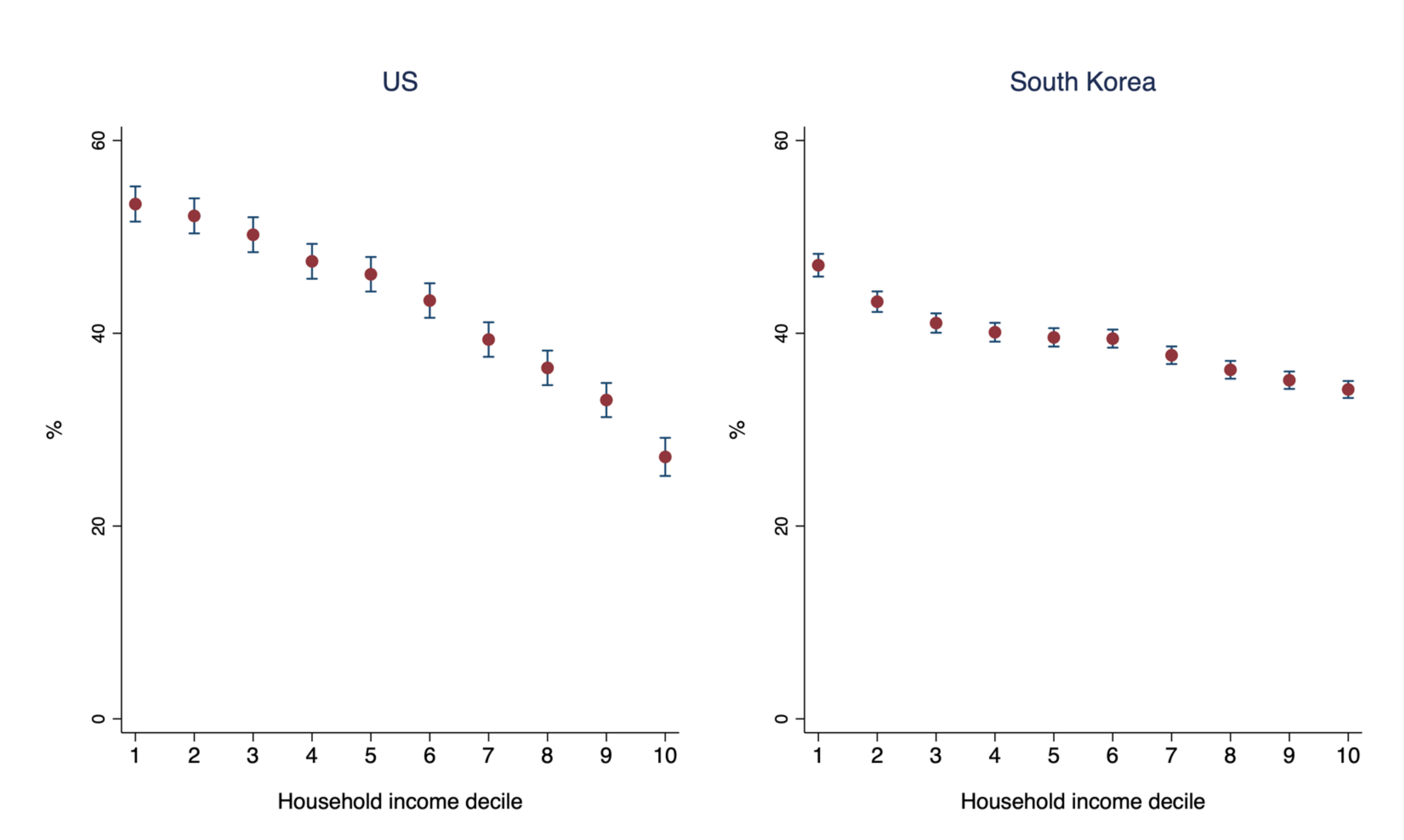

© 2026 Park S et al. *JAMA Health Forum*.

Panel B. Current smoker

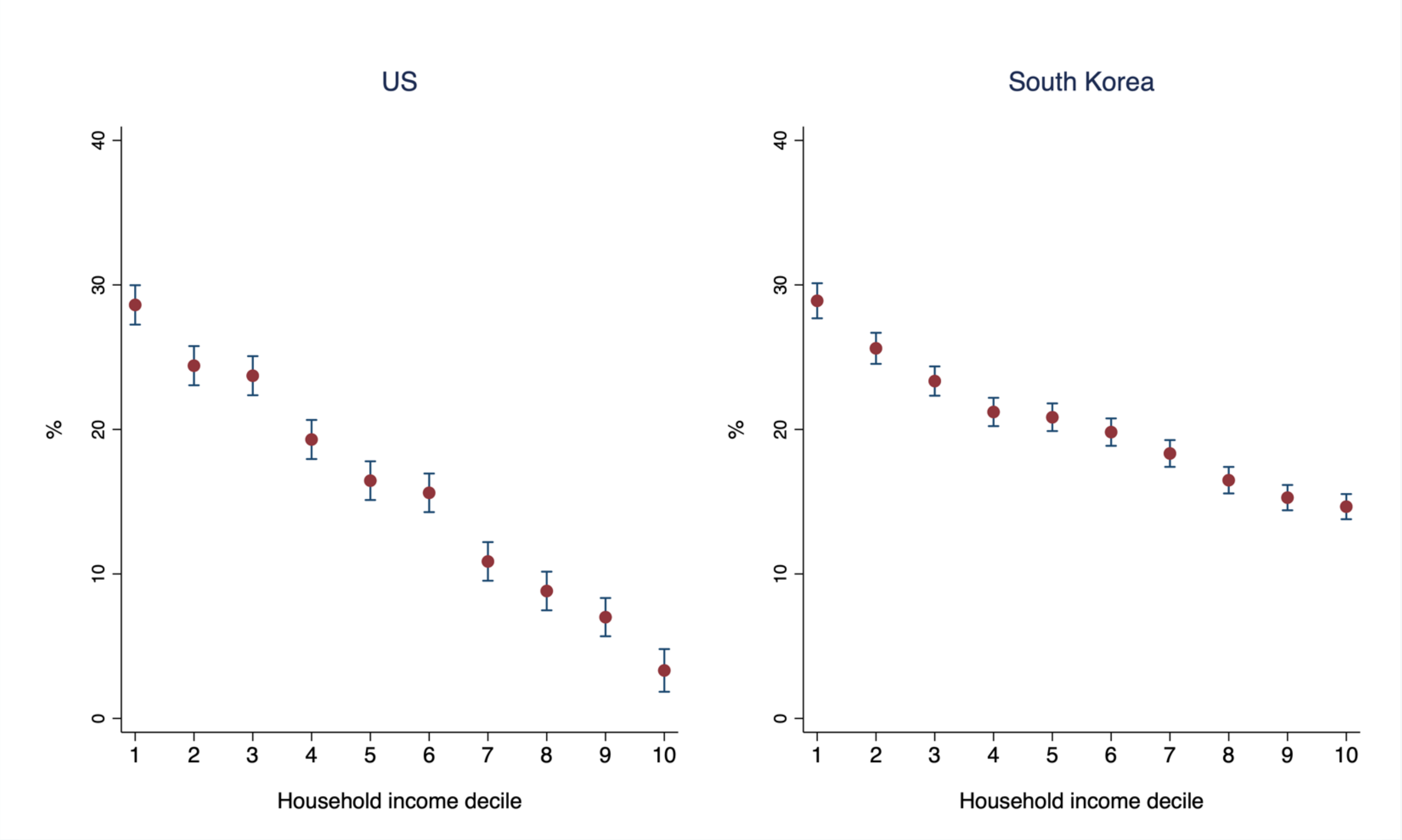

© 2026 Park S et al. *JAMA Health Forum*.

Panel C. Overweight

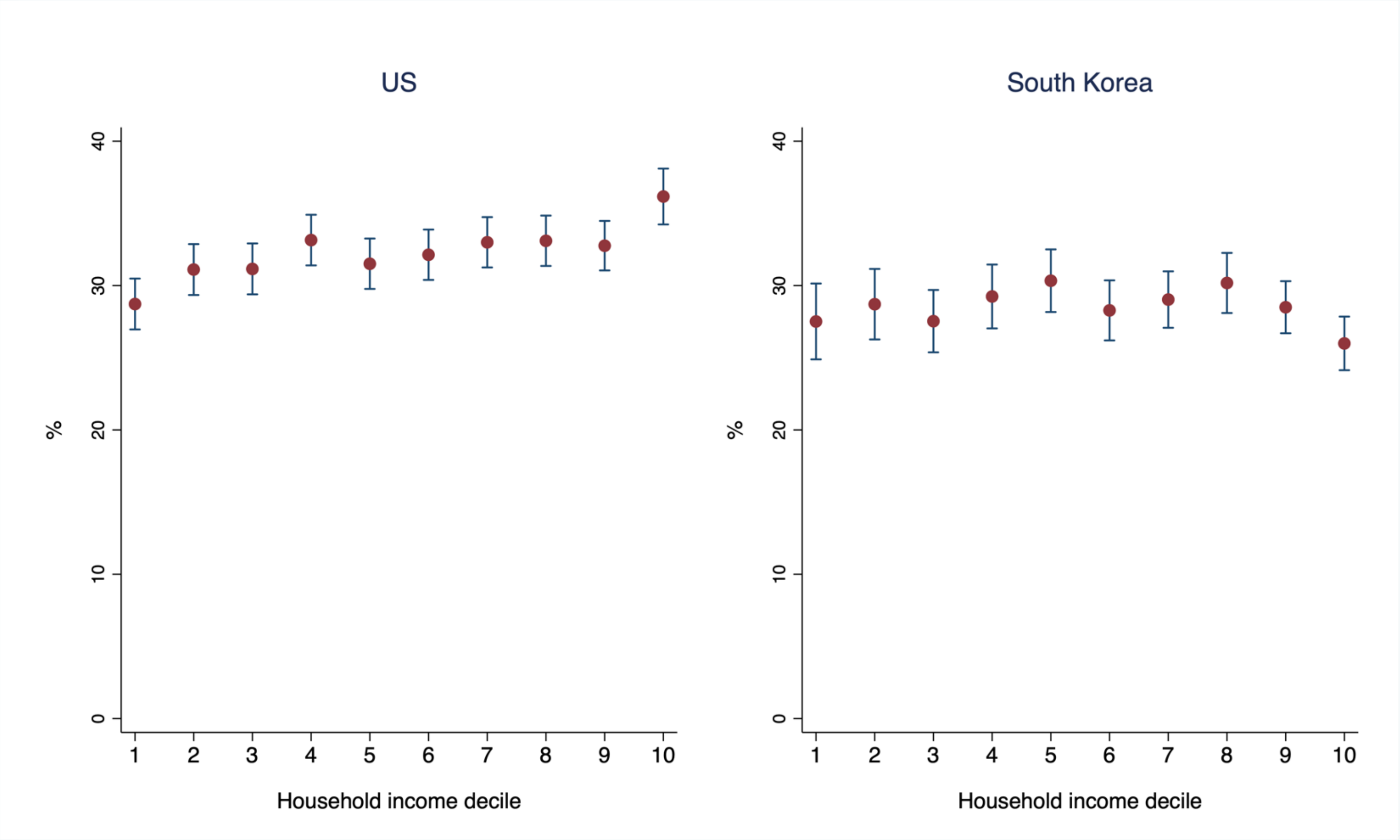

© 2026 Park S et al. *JAMA Health Forum*.

Panel D. Excessive drinking

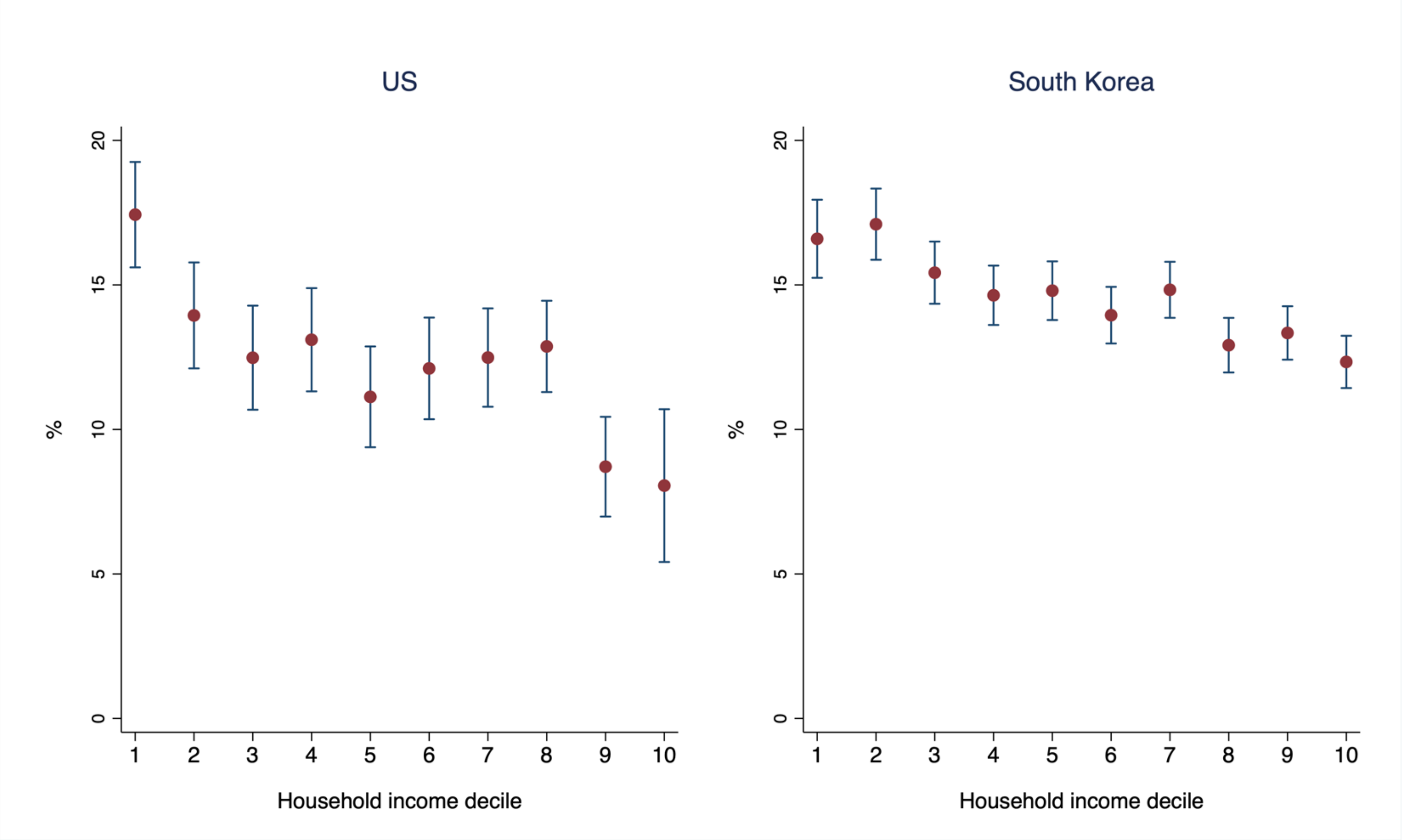

© 2026 Park S et al. *JAMA Health Forum*.

eFigure 6. Trends in clinical outcomes by household income decile

Panel A. Uncontrolled hypertension (systolic)

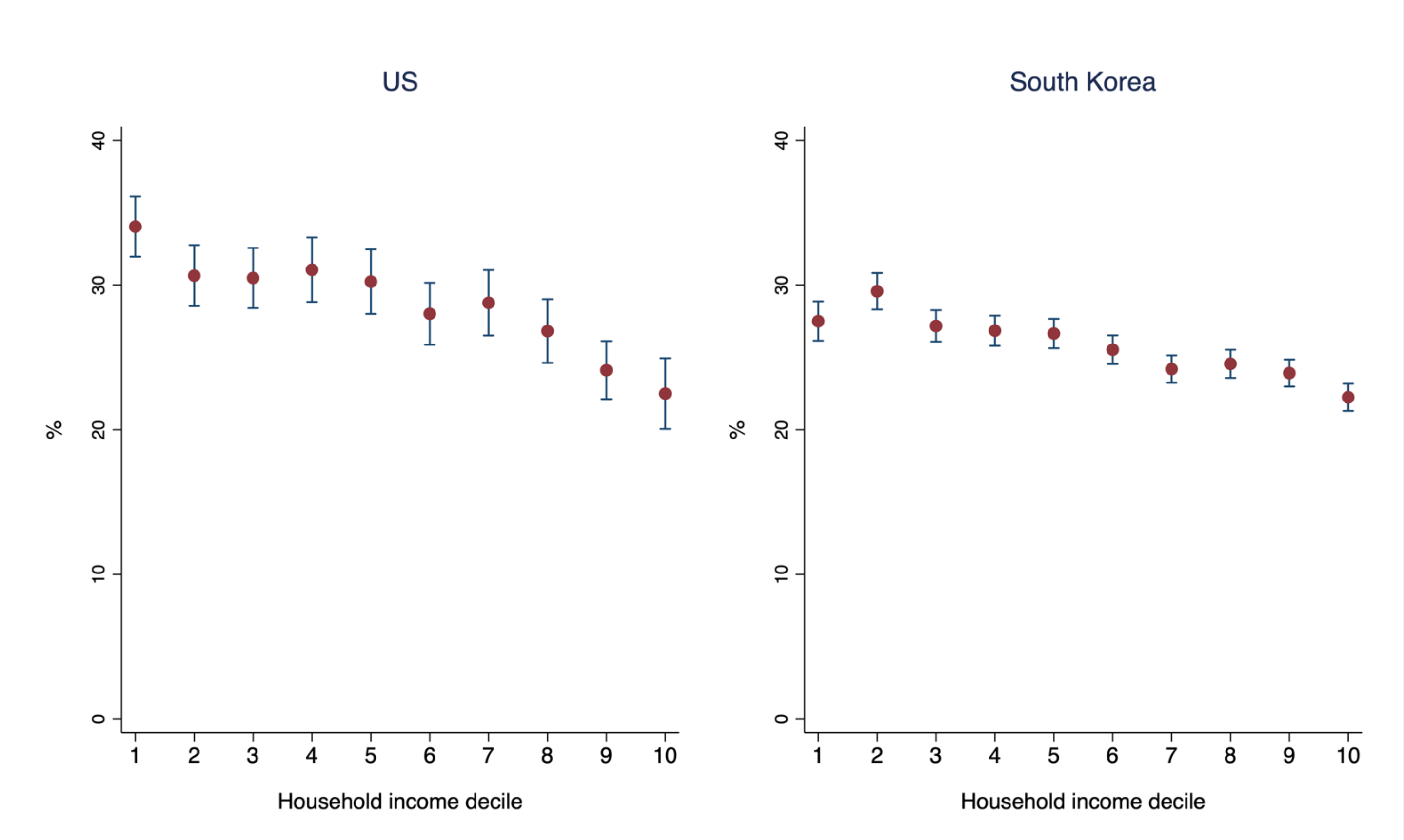

© 2026 Park S et al. *JAMA Health Forum*.

Panel B. Uncontrolled hypertension (diastolic)

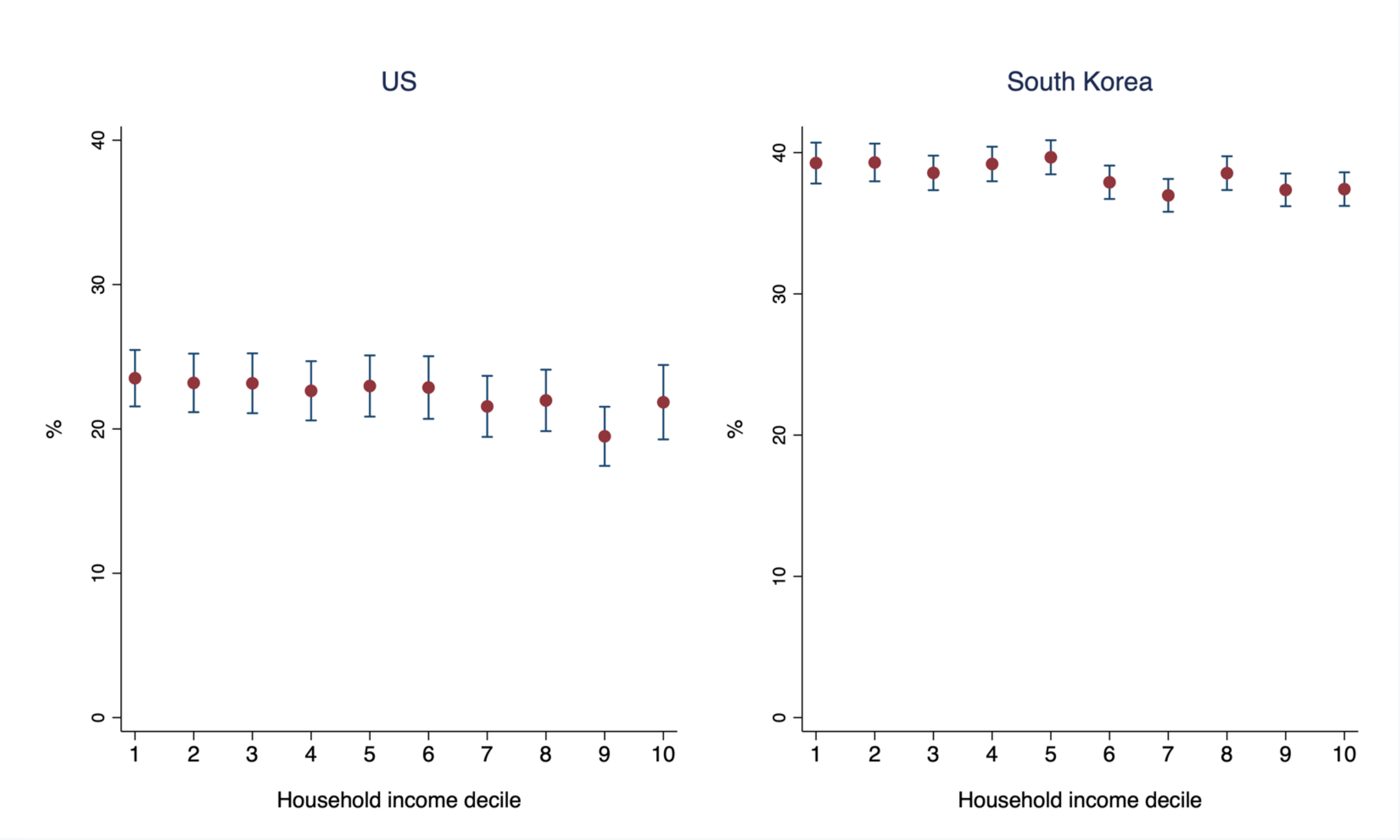

© 2026 Park S et al. *JAMA Health Forum*.

Panel C. Uncontrolled diabetes mellitus

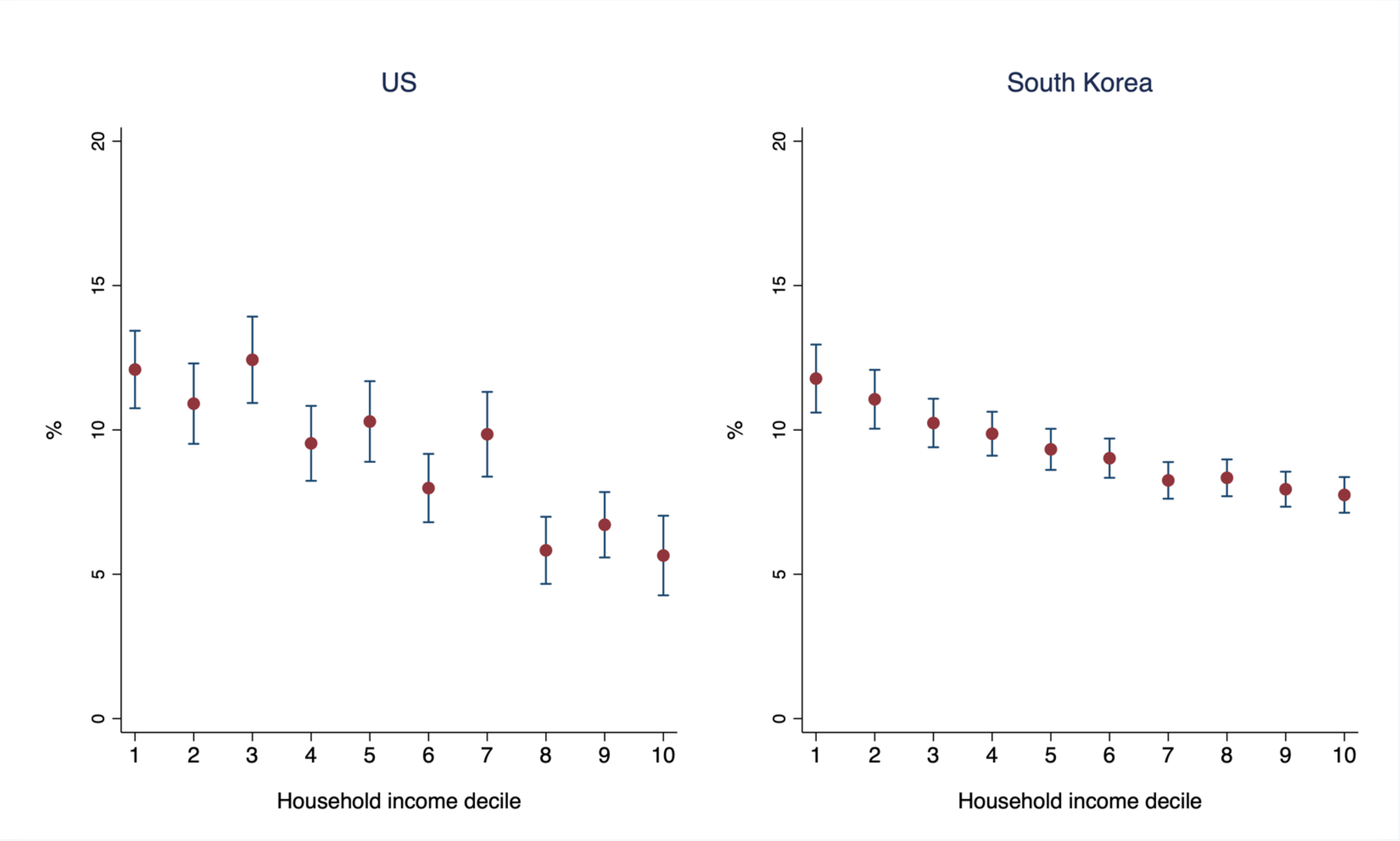

© 2026 Park S et al. *JAMA Health Forum*.

Panel D. Low HDL-cholesterol

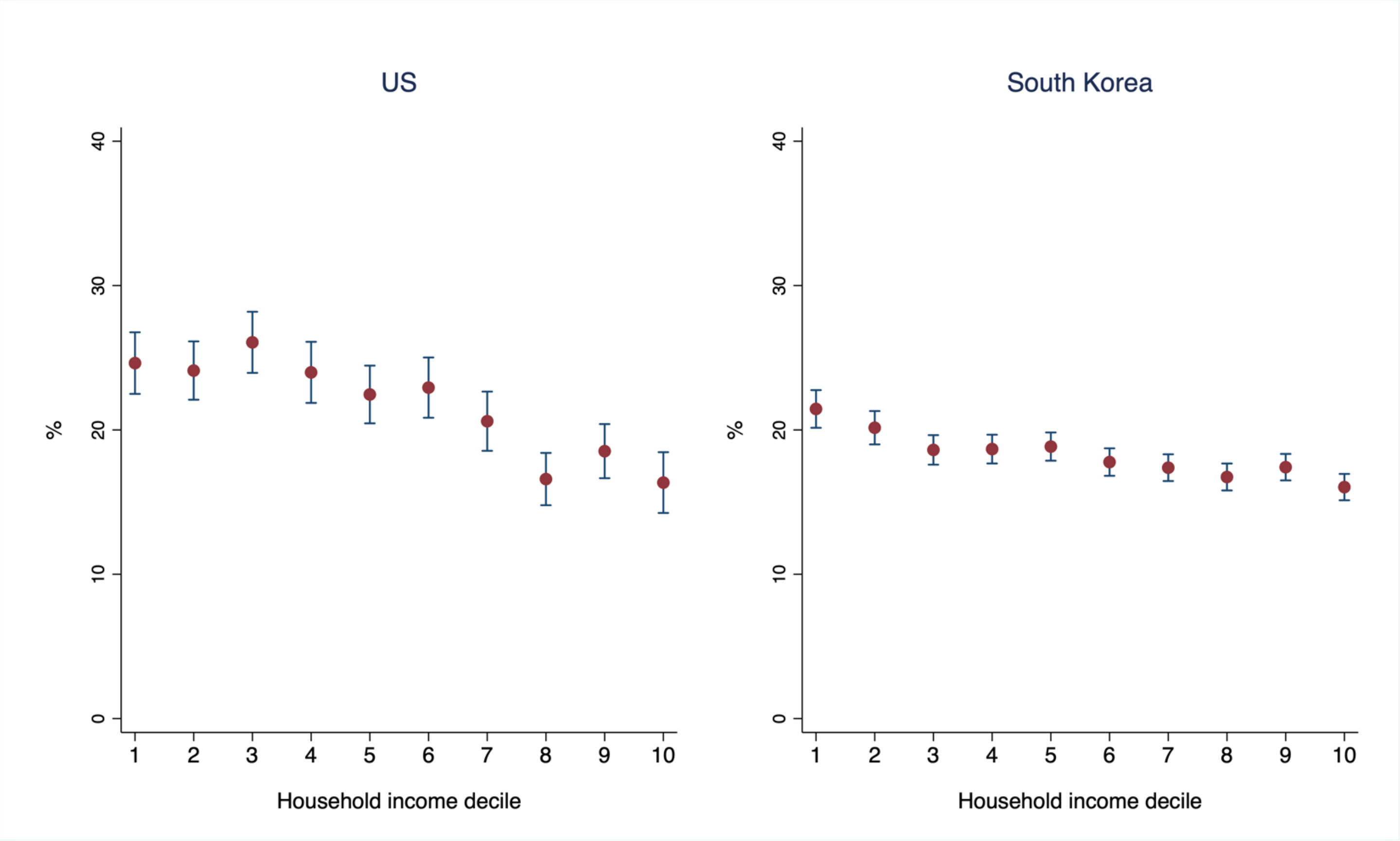

© 2026 Park S et al. *JAMA Health Forum*.

Panel E. Elevated LDL-cholesterol

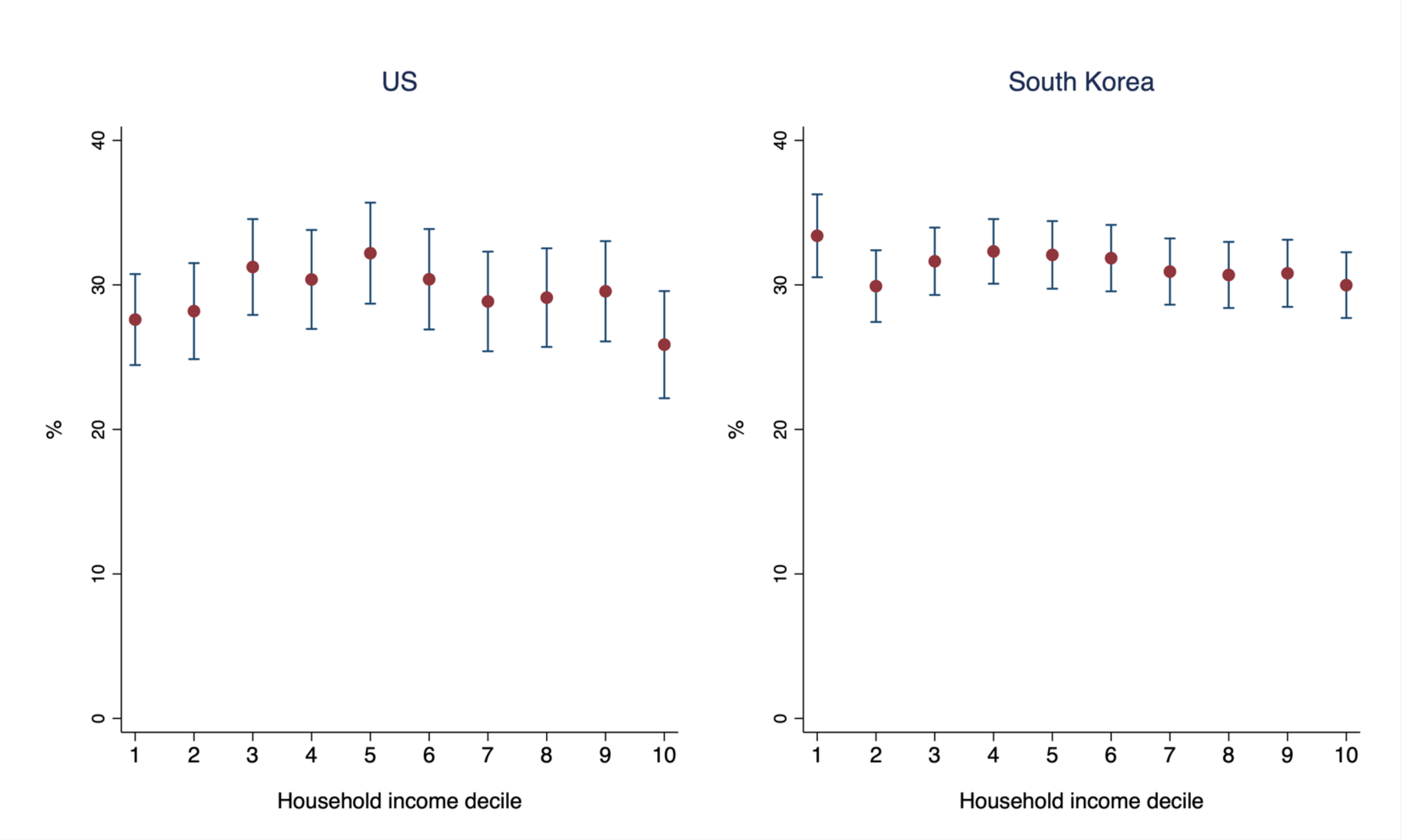

© 2026 Park S et al. *JAMA Health Forum*.

Panel F. Hypertriglyceridemia

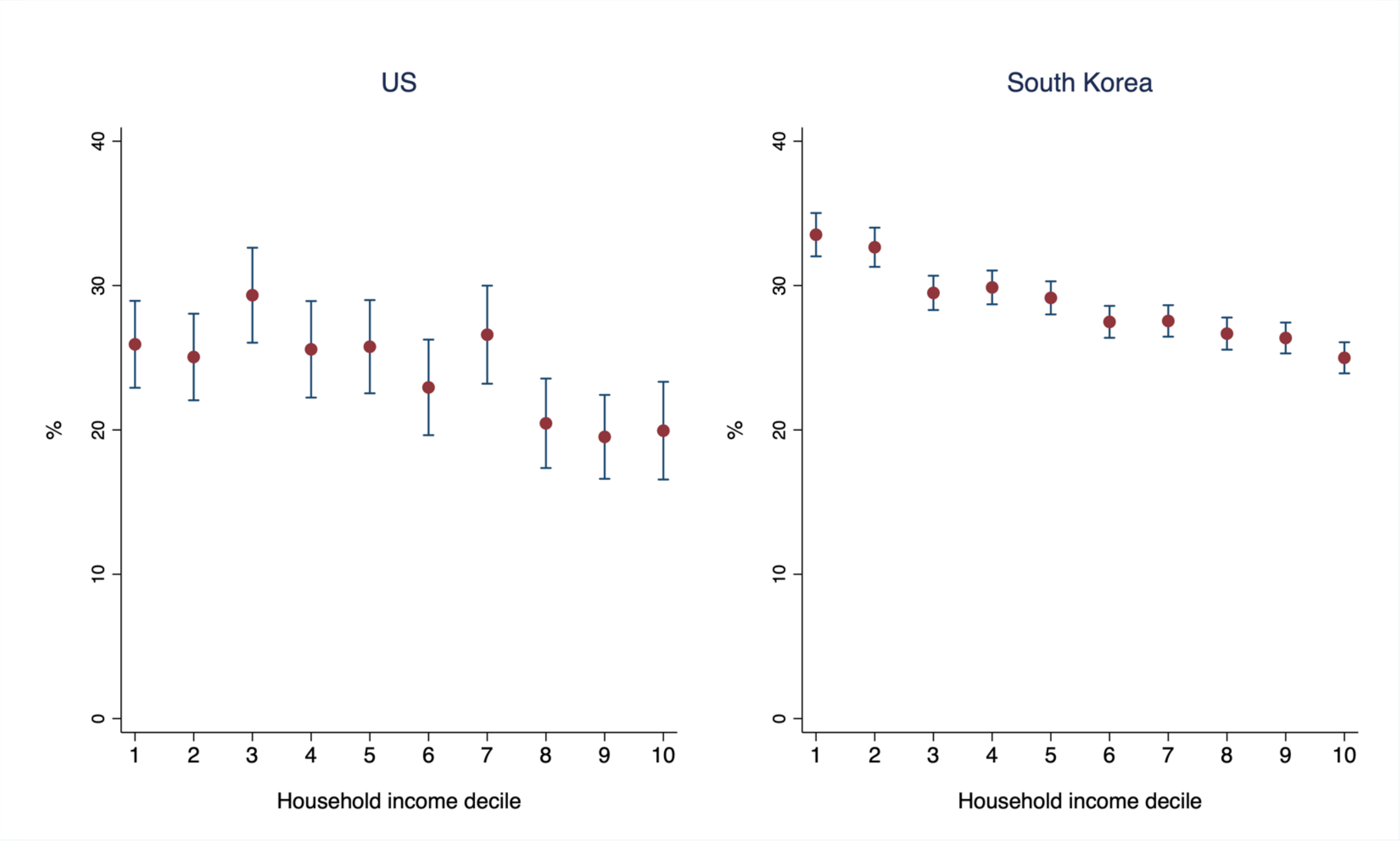

© 2026 Park S et al. *JAMA Health Forum*.

© 2026 Park S et al. *JAMA Health Forum*.
