# Supplementary material for: Income-Based Inequalities in Health System Performance in the US and South Korea
Source: JAMA Health Forum. 2026 Mar 20;7(3):e260136. doi: 10.1001/jamahealthforum.2026.0136 (PMC13005164; doi:10.1001/jamahealthforum.2026.0136)
Supplement: Supplement 2. — Data Sharing Statement [file jamahealthforum-e260136-s002.pdf]

Data Sharing Statement

Park. Income-Based Inequalities in Health System Performance in the US and South Korea. *JAMA Health Forum*. Published March 20, 2026. doi:10.1001/jamahealthforum.2026.0136

Data
Data available: No

Additional Information
Explanation for why data not available: The data is publicly available, but we can share our own dataset upon request.
